# High alert drugs screening using gradient boosting classifier

**DOI:** 10.1038/s41598-021-99505-4

**Published:** 2021-10-11

**Authors:** Pakpoom Wongyikul, Nuttamon Thongyot, Pannika Tantrakoolcharoen, Pusit Seephueng, Piyapong Khumrin

**Affiliations:** Department of Family Medicine, Faculty of Medicine, Biomedical Informatics Center, Chiang Mai University, Chiang Mai, Thailand

**Keywords:** Health care, Drug regulation

## Abstract

Prescription errors in high alert drugs (HAD), a group of drugs that have a high risk of complications and potential negative consequences, are a major and serious problem in medicine. Standardized hospital interventions, protocols, or guidelines were implemented to reduce the errors but were not found to be highly effective. Machine learning driven clinical decision support systems (CDSS) show a potential solution to address this problem. We developed a HAD screening protocol with a machine learning model using Gradient Boosting Classifier and screening parameters to identify the events of HAD prescription errors from the drug prescriptions of out and inpatients at Maharaj Nakhon Chiang Mai hospital in 2018. The machine learning algorithm was able to screen drug prescription events with a risk of HAD inappropriate use and identify over 98% of actual HAD mismatches in the test set and 99% in the evaluation set. This study demonstrates that machine learning plays an important role and has potential benefit to screen and reduce errors in HAD prescriptions.

## Introduction

Drug prescription is a process involving legally dispensing medicine as prescribed by a medical doctor. Several factors are considered in this process including patient symptoms, underlying diseases, route of administration, drug potency and efficacy to obtain optimal patient outcomes. In the outpatient department at the Maharaj Nakorn Chiang Mai Hospital, the drug prescription process starts when a doctor enters input information including type and dosage of medication into the electronic health record systems (EHR). The prescribed drug is then double-checked by a pharmacist before being dispensed to a patient. The process of drug prescription for an inpatient is different. The doctor needs to write the prescription order on a paper form and also enter the same information into EHRs. Both information sources are again checked by a pharmacist. The pharmacist checks that the information on the paper form is exactly the same as the electronic prescription in EHRs, especially the dosage, route of administration, and frequency of use. After the prescription is verified, a nurse collects the drugs and triple-checks the request information to ensure that the drugs are dispensed to the correct patient with the right route, dose, and timing. Multiple checks during the prescription process are designed to reduce human errors. However, errors still persist even with this rigorous checking process.

A previous study in the U.S.A. reported that medical errors are one of the ten most common causes of death^1^. Drug prescription errors account for 70% of medical errors, most commonly occurring within hospitals. These errors can lead to serious complications for patients^1–4^ which may be fatal^5, 6^. For instance, a patient might receive drugs that they are allergic to or receive drugs that interfere or interact with each other. A 2014 study in a Thai tertiary hospital^6^ reported that the incidence of drug prescription errors was high (2.25% compared to total verified drugs). Surprisingly, High Alert Drugs (HAD) accounted for 3.69% of the drug prescription errors. Common causes of drug prescription errors include human input error (e.g., poor legibility of handwriting^5^, substandard medical abbreviation use^7^), drug dispensation errors (e.g. expired drugs, wrong administration technique, wrong time or frequency, wrong route of administration, wrong patient), and drug administration errors (e.g. administration of a non-prescribed drug, inappropriate dose, drug to drug interactions, contraindicated or history of drugs allergy in the patients, inappropriate drugs, drugs that are no longer recommended for use)^5, 8^.

Pharmacists and nurses play a major role in reducing drug administration errors. They are involved in the checking steps of drug verification to reduce drug dispensation and administration errors, especially in a large healthcare center where the care of patients in an inpatient department is very complex. Although there are several verification processes, the number of drug prescription errors is still high according to the evidence as mentioned above. Therefore, an electronic system was developed to minimize the errors by using computerized physician order entry (CPOE), defined as “a use of computer assistance to directly enter medication orders from a computer or mobile device by any licensed healthcare professional”^9^. Previous research presented that the implementation of CPOE significantly decreased input errors compared with the traditional drug prescription method. However, there was no significant reduction in adverse drug events (ADE)^10^. This result indicated that ADE is a complex problem for which digital input transformation alone is not sufficient to solve the problem. Therefore, medical institutes introduced computerized clinical decision support systems (CDSS), which are systems enhancing medical decisions with targeted clinical knowledge, patient information, and other health information alongside CPOE to improve patient safety^11^.

In the last decade, healthcare professionals have been attempting to lower ADEs. New policies such as the American Society of Health System Pharmacists (ASHP) guidelines on preventing medication errors in hospitals^12^ were published by the American Society of Health-System Pharmacists aiming to improve and prevent medication errors. These guidelines provide a modification of the joint commission’s medication management system, comprising of 11 steps for managing a healthcare system to prevent and mitigate patient harm from medication errors during drug prescription processes. Although the additional guidelines were designed to help medical professionals to effectively and practically manage drug prescription processes and minimize medication errors, it created extra work to accomplish the goal. Pharmacists play a key role in these extra steps but it leads to a bottleneck effect. To achieve a more effective solution means more human resources and time are required. Computerized systems seem to have a potential role to reduce this bottleneck effect. Nowadays, machine learning has been introduced into many domains and increasingly within the medical field. Reduction in human resources and time and increasing work outcome accuracy are major advantages of machine learning models which perfectly fit the requirements to solve the ADE problems. Prior research showed that the implementation of CDSSs has a potential benefit for electronic prescriptions to improve the quality of care, to reduce medication errors, and to potentially decrease long-term health care costs^8, 13–16^. A meta-analysis of controlled clinical trials showed that implementation of CPOE with CDSS appeared to achieve small to moderate improvement^17^. However, the design of a CDSS is still a challenge, with a trade off between system reliability and cost of investment, conferring large improvement in the quality of care while also balancing the threat of alert fatigue or burn out which is open for further research^17, 18^. We reviewed within our scope of interest some prior research of CDSS implemented for ADE solutions. MedAware, a CDSS utilized with a machine learning model, was developed to screen for medication errors. The MedAware system was tested on a large outpatient data set and the evaluation result demonstrated 76.2% of alerts were found to have identified potential medication errors^19^. Other research showed that machine learning models had significantly better predictions of appropriate warfarin dosing than other statistical methods^20, 21^. Apart from the scope of reducing errors in medication, Szlosek and his colleagues tested machine learning models against a manual review process for evaluating mild traumatic brain injury to support the decision making for ordering a CT scan. The automated process showed an important role in this process because it reduced the extensive amount of time for a trained medical practitioner to make a decision. The results showed that the machine learning model performance was better than the traditional process^22^. These research examples show the potential power of a machine learning approach on drug verification processes not only in predicting a final decision but in developing systems capable of recommending alternative possible solutions and explaining evidence supporting the decision^23, 24^ which may help to improve reducing ADEs and diminishing human resources or time-consuming processes while achieving the goal of effective care.

Investigating machine learning driven CDSS for a potential solution for drug prescription errors, some prior research had focused on developing new approaches to utilizing machine learning models to screen drug prescription errors, in particular with high alert drugs (HAD)^20, 21^. Compared with other ordinary drugs, HADs carry a greater risk of ADEs and severe consequences which hospital administrators must monitor closely^25^. Recent studies showed that up to 50% of ADEs are related to HADs^26^. Moreover, ADEs occurring from HAD related to a high mortality due to a narrow drug therapeutic window^26^. In August 2020, Chun-Tien Tai and colleagues used machine learning to predict the risk for digoxin treatment^27^. The results showed that the best model performance was successfully able to identify the risk (0.852 precision and 0.742 recall). This study confirmed that machine learning techniques can yield favorable prediction effectiveness for HAD medication treatment, thereby decreasing the risk of ADEs and improving medication safety^27^. Therefore, HAD prescription errors are a serious issue in a hospital while the utilization of machine learning models has a potential role to minimize this problem. As a result, we intentionally designed a new approach that utilizes machine learning models to predict the appropriateness of HAD use for an individual visit and we believe that this research could bring potential benefits to the research community and help to reduce ADEs caused by HAD while saving human resources and time.

## Materials and methods

### Population

We retrospectively collected patient data from outpatient departments (OPD) and inpatient departments (IPD) at the Maharaj Nakorn Chiang Mai Hospital in 2018, including visit numbers (TXN) (integer: a unique identifier of an individual visit), gender (category: male and female), age (integer: year), the International Statistical Classification of Diseases and Related Health Problems 10th Revision (ICD10) code (category: 3,280 unique ICD10 codes in the OPD data set and 2,020 unique ICD10 codes in the IPD data set), and drug prescription (category: 1,184 drug codes in the OPD data set and 1,767 unique drug codes in the IPD data set). The drug prescription data were used as a target label.

### Data preprocessing

The gender, ICD10, and drug prescription features were transformed by applying one-hot encoding technique and then aggregated by TXN (representing one data instance as a hospital visit). The number of total drugs (integer) per TXN was calculated and added as a new feature to the aggregated data set. The aggregated data set was split for two development cycles: HAD binary classification and HAD type classification. In the HAD binary classification cycle, the drug prescription features were consolidated into two subgroups: visits without high alert drugs prescriptions (non-HAD) and visits with high alert drugs prescriptions (HAD). Visits prescribing at least one HAD drug from the HAD drug list presenting in Table 2 were categorized as HAD; otherwise non-HAD. In the HAD type classification cycle, we used the trained machine learning model from the HAD binary classification cycle to predict the probability of HAD use of the data sets in this cycle (float: probability of HAD use) and used the prediction results to filter visits with low probability of HAD use from the data set. The prediction results were added to the selected data as a new feature in this cycle. The drug prescription features were categorized into seven subgroups (category) including six HAD groups (see the full list of drugs in each HAD type in Table 3) and one non-HAD group in accordance with the Pharmacy division of Maharaj Nakorn Chiang Mai Hospital. Then, the HAD subgroups were assigned to the selected data as a target label. The summary of input features and labels of data sets used in both cycles is shown in Table 1. Basic statistical parameters (count, average, standard deviation) were used to analyze the distribution of the data and HAD types. Lasso linear model^28^ was used to calculate the feature relevance. The processes of computational execution were run in Python 3.7.4 using scikit-learn version 0.22.1 on Google co-laboratory locally hosting on an Intel(R) Xeon(R) CPU E5-2650 v4 @ 2.20GHz 2.20 (2 processors) with 64 GB RAM. The study was carried out in accordance with guidelines of the Faculty of Medicine, Chiang Mai University (Study code: PHY-2562-06160). The study was approved by the Research Ethics Committee of Faculty of Medicine, Chiang Mai University. All methods were carried out with exemption criteria (waiver of informed consent) and the need for informed consent was waived by the Research Ethics Committee of Faculty of Medicine, Chiang Mai University in accordance with the Faculty of Medicine, Chiang Mai University Ethics regulations.

**Table 1:** Input features and labels of data sets.

Cycle 1	
Input features	Labels
Male gender (binary: yes or no)	Non-HAD
Age (integer: year)	HAD
ICD10 (binary: prescribed and not prescribed)	
Total drugs (integer)	
Cycle 2	
Input features	Labels
Male gender (binary: yes or no)	Non-HAD
Age (integer: year)	ANS
ICD10 (binary: prescribed or not prescribed))	BIG
Total drugs (integer)	CVS
Probability of HAD binary classification (float)	CNS
	END
	Tumor

**Table 2:** High alert drug (HAD) groups.

HAD groups	Drug names
General HADs	Norepinephrine, Dopamine, Dobutamine, Calcium gluconate, Digoxin, Isoproterenol, Warfarin, Magnesium sulfate, Heparin, Potassium chloride, Regular insulin, Adrenaline
Narcotics and psychotropic drugs	Ketamine, Midazolam, Pethidine, Alprazolam, Phentermine, Oxycodone, Ephedrine, Nitrazepam, Fentanyl, Methylphenidate HCL, Methadone, Pseudoephedrine, Zolpidem, Morphine
Intravenous cytotoxic chemotherapy drugs	5-Fluorouracil, Arsenic trioxide, Azacitidine, Bendamustine, Bleomycin, Busulfan, Cabazitaxe, Carboplatin, Carmustine, Cisplatin, Clofarabine, Cyclophosphamide, Cytarabine, Dacarbazine, Dactinomycin, Decitabine, Docetaxel, Doxorubicin, Epirubicin, Eribulin mesylate, Etoposide, Fludarabine, Gemcitabine, Idarubicin, Ifosfamide, Irinotecan, Ixabepilone, L-Asparaginase, Liposomal-doxorubicin, Melphalan, Methotrexate, Mitomycin C, Mitoxantrone, Oxaliplatin, Paclitaxel, Paclitaxel (polymeric micelle), Pemetrexed, Topotecan, Vinblastine, Vincristine sulfate, Vinorelbine
Oral cytotoxic chemotherapy drugs	Temozolomide, Methotrexate, Melphalan, Tegafur-Uracil, Tegafur-Gimeracil-Oteracil, Cyclophosphamide, Capecitabine, Hydroxyurea, Fludarabine phosphate, Etoposide, Topotecan, Mercaptopurine, Vinorelbine, Thioquanine, Chlorambucil

### Machine learning algorithm selection

Before starting the first cycle, we ran three folds cross-validation with Dummy classifier, Decision tree classifier, Stochastic gradient descent (SGD) classifier, Kneighbor classifier, Gaussian process classifier, Gaussian Naive Bayes, Random forest classifier, and Gradient boosting classifier to internally validate the model with the OPD and IPD data sets from the HAD binary classification cycle to find the best model for the experiment. We evaluated the model performance using average accuracy and determined the best model. Grid search was applied to find the optimal parameter setting of the model.

### Cycle 1: HAD binary classification

Regarding the definition of HAD, we labeled visits that were prescribed with at least one medication of any type of HAD as an actual HAD visit. On the other hand, visits that were prescribed with other medications without HAD were labeled as a non-HAD visit. The data sets were shuffled with seed and fixed split into training and test sets with 75:25 ratio. Then, we separately trained the selected machine learning model with the OPD and IPD training data sets and validated the model with the corresponding test sets. The results of model prediction were presented in a form of probability of a HAD prescription. A probability value of 1 represented those visits that were most likely to receive HAD and zero probability represented visits with least likely chance of HAD prescription. We then analyzed the HAD prediction results on different probability numbers to identify an appropriate cut point of HAD prediction. If the prediction value was above the cut point, the visit was classified as predicting a HAD visit. If the value was equal or below the cut point, it was classified to predict a non-HAD visit. The best cut point was considered from the highest number of the screening index (S-index) (see Eq. 1). The S-index was calculated by F1-score and recall in which the best score refers to the best balance of those two parameters. Recall was counted in the equation because we aim to minimize the number of false negative in order to avoid missing an event of inappropriate HAD use. Finally, the trained model with the selected cut points was evaluated with the test sets in the HAD type classification cycle.

1 $$\begin{aligned} \begin{aligned} S{\text{- }}index = F1{\text{- }}score \times Recall \end{aligned} \end{aligned}$$

#### HAD binary classification

We calculated F1-score and recall and used these parameters to calculate S-index on a moving cut point range from 0 to 1. Then, we selected the best cut point by the highest number of S-index. The selected cut point was rounded down to two decimal points. The selected cut point was used to predict non-HAD and HAD. Then, the model performance was validated and reported with accuracy, precision, recall, and F1-score.

#### Post-hoc result readjustment

After the model was initially validated, we then systematically reviewed the prediction results using HAD percent to adjust false positive, false negative, true positive, and true negative and calculated specific false negative values. The readjustment step helped us to filter unusual harmless prescriptions from actual inappropriate HAD uses.

##### HAD percent

HAD percent is the proportion of visits that were prescribed HADs relative to all visits with the same diagnosed ICD10 (see Eq. 2). High HAD percent indicates that the ICD10 has high frequency use of HAD which could be interpreted that HAD use is most likely appropriate. For example, I342 (non-rheumatic mitral valve stenosis) has a HAD percent of 92% which is interpreted as 92% of visits with this ICD10 were prescribed with HAD. We selected a default threshold of 50% where HAD percent equal or higher than the threshold implies ICD10s with high HAD use. Conversely, a value that is less than the threshold is classified as ICD10s with low HAD use. False positive and false negative were adjusted by this HAD percent indicator. We applied this indicator to verify the appropriateness of HAD use in visits that contained both single and multiple ICD10s. The HAD pattern of single and multiple ICD10 per visit is raised later in the discussion section of this paper in terms of one ICD10 per visit and multiple ICD10s per visit. The 400,000 prescriptions with ICD10 records were randomly sampled from each OPD and IPD data set and used for calculating HAD percent separately for those two data sets. If one visit only contained one ICD10, we directly use the HAD percent of the ICD10 to interpret the HAD use of the visit. In cases of multiple ICD10s per visit, if visits contained at least one ICD10 with high HAD percent, we interpreted those visits as related to high HAD use. Conversely, if visits contained no ICD10 with high HAD percent, we interpreted those visits as related to low HAD use.

2 $$\begin{aligned} \begin{aligned} HAD\ percent^{\textit{i}} = \frac{HAD\ visit\ of\ ICD\textit{10}^{\textit{i}}}{Total\ visit\ of\ ICD\textit{10}^{\textit{i}}} \end{aligned} \end{aligned}$$

##### Adjusted false and true positive (aFP & aTP)

According to HAD percent, adjusted false positive (aFP) is the number of false positives deducted by false positive visits with the HAD percent above or equal to 50% (see Eq. 3). Adjusted false positive is visits that are predicted to high HAD use but do not actually use HAD and present with low HAD percent. The aFP visits show that the evidence of absent actual HAD prescription and low HAD percent indicate less likely chance of HAD use which is opposite to the model prediction. This pattern of mismatch was used to measure the level of the model prediction performance. A lower number of adjusted false positive indicates a better model. On the other hand, adjusted true positive (aTP) is the true positives and false positives with high chance of HAD use (HAD percent above or equal to 50%) (see Eq. 4).

3 $$\begin{aligned}&aFP = FP - FP_{\textit{HAD percent}\ge 50\%} \end{aligned}$$

4 $$\begin{aligned}&aTP = TP + FP_{\textit{HAD percent}\ge 50\%} \end{aligned}$$

##### Adjusted false and true negative (aFN & aTN)

In contrast to adjusted false positive, adjusted false negative (aFN) is the number of all false negatives deducted by false negative visits with the HAD percent lower than 50% (see Eq. 5). The remaining false negative visits after deduction (aFN) contain visits with high HAD percent which indicate that those visits are highly likely to relate to HAD regarding high HAD percent and actually prescribed with HAD but the model suggested low HAD association. Visits with high HAD percent ICD10 and actually use of HAD in contrast with low prediction of HAD use were also used as an indicator for a prediction error. Visits with actual HAD prescription but low HAD prediction and low HAD percent were explored for HAD - ICD10 mismatch. Adjusted true negative (aTN) is the true negatives and false negatives with low chance of HAD use (HAD percent below 50%) (see Eq. 6).

5 $$\begin{aligned}&aFN = FN - FN_{{\textit{HAD percent}} <50\%} \end{aligned}$$

6 $$\begin{aligned}&aTN = TN + FN_{{\textit{HAD percent}}<50\%} \end{aligned}$$

##### Specific false negative ratio (sFNR)

Specific false negative ratio (sFNR) is a modified false negative ratio measured for each ICD10 (see Eq. 7). The specific false negative ratio was used to measure how well the model is able to detect false negatives for each ICD10 specifically.

7 $$\begin{aligned} \begin{aligned} sFNR_{\textit{i}} = \frac{FN\ of\ ICD\textit{10}_{\textit{i}}}{Total\ visits\ of\ ICD\textit{10}_{\textit{i}}} \end{aligned} \end{aligned}$$

### Cycle 2: HAD type classification

In this cycle, the classification labels were broken into six HAD types of Autonomic Nervous System (ANS), Blood Inflammation Gout (BIG), Cardiovascular System (CVS), Central Nervous System (CNS), Endocrine (END), Tumor (Tumor), as classified by Bertram G. Katzung Basic and Clinical Pharmacology edition 14th 2017^29^ (see the list of drugs on each HAD type in Table 3), and one non-HAD. The prediction output presented a probability range from 0 to 1 for each of seven labels, consisting of six HAD types and one non-HAD type, for each visit.

**Table 3:** HAD types.

HAD types	Drug groups	Drug names
ANS	Adrenergic agonist, Decongestant, Sympathomimetic drugs	Dobutamine, Dopamine, Isoprenaline, Isuprel, Pseudoephedrine, Ephedrine, Levophel, Methylphenidate, Norepinephrine, Adrenaline
BIG	Anticoagulant	Befarin, Heparin, Moforan, Orfarin
CVS	Antiarrhythmic drugs, Cardiac glycosides	Magnesium Sulfate, Magfifty, Potassium Choride, Digoxin
CNS	Anesthetic drugs, Opioid, Sedative drugs, Hypnotic drugs	Ketamine, Fentanyl, Methadone, Morphine, Oxycodone, Pethidine, Alprazolam, Midazolam, Xanax, Zolpidem
END	Antidiabetic drugs, Bone hemostasis	Actrapid, Humulin, Insugen, Calcium Gluconate
Tumor	Antibiotics, Alkylating agents, Anti-Inflammatory drugs, Anti-metabolites, Microtubule inhibitor, other	Pharmorubicin, Lipo-Dox, Adriblastina, Adrim, Mitomycin, Mitoxantrone, Vasimycin, Zavedos, Bleocin, Alkyloxan, Alkeran, Bicnu, Busulfex, Cycloxan, Dacarbazine, Eloxatin, Endoxan, Holoxan, Kemocarb, Cisplatin, Leukeran, Oxalip, Oxitan, Paraplatin, Ribomustin, Temodal, Methotrexate, Alimta, Dacogen, Effcil, Emthexate, Evoltra, Gemzar, Gemita, Hydrea, Intacape, Puri-Nethol, Purinetone, Thioguanine, Ts-One, Ufur, Vidaza, Xeloda, Alimta, Cytosar, Cytarine, Dactinomycin, Lanvis, Fludara, Daxoel, Docetaxel, Anzatax, Halaven, Intaxel, Ixempra, Jevtana, Navelbine, Paxus Pm, Taxotere, Vinblastin, Vincristine, Vinelbine, Asadin, Campto, Cisplatin, Fytosid, Hycamtin, Irinotel, Lastet, Leunase

#### HAD filter

The selected cut points and trained machine learning models from the HAD binary classification cycle were used in this process. The models were used to predict the probability of HAD use for the OPD and IPD data sets. Visits with the probability of HAD use above the selected cut points were included for analysis in this cycle and visits with the probability of HAD use below or equal to the selected cut points were excluded. The excluded data were used to evaluate the model performance derived from the HAD binary classification cycle.

#### HAD type classification

The included OPD and IPD data sets were shuffled with seed and fixed split into a training and test sets with 75:25 ratio. The Gradient boosting classifier was trained with the training set and validated with the test set. The classification results presented the probability of HAD use. For each HAD type, we selected two cut points at 25th percentile (P25) and 75th percentile (P75), calculated from all predictions of the test sets, to classify HAD use in accordance with three rules.

1. Prediction value less than or equal to P25 is interpreted as no use of that HAD type.
2. Prediction value between P25 and P75 is an uncertainty zone which is excluded from HAD interpretation.
3. Prediction value of more than P75 is interpreted as the use of corresponding HAD type.

Table 4 shows an example of how HAD use is classified and the probability of HAD prediction for each type compared with actual HAD type (shown in the ANS, BIG, CNS, CVS, END, Tumor and non HAD columns). The probability with asterisk represents the number that is classified as HAD use (above P75) and matches with the actual drug prescription (marked with asterisk) in the Drugs column.

**Table 4:** Model prediction of HAD types.

ICD10s	Drugs	ANS	BIG	CNS	CVS	END	Tumor	non HAD
Acute nasopharyngitis	Beramol, Fenafex, Tussis, Pseudoephedrine*	0.28*	0.02	0.01	0.00	0.00	0.01	0.68
Paroxysmal atrial fibrillation	Maforan*	0.04	0.17*	0.01	0.01	0.00	0.02	0.75
Bladder cancer	Morphine*, Kapanol, Gabapentin	0.01	0.01	0.35*	0.00	0.00	0.01	0.62
Atrial septal defect	Sildenafil, Digoxin*, Spironolactone	0.02	0.02	0.03	0.46*	0.00	0.01	0.46
Rheumatoid arthritis	*(d) Emthexate*, Folivit, Caltab	0.02	0.03	0.01	0.01	0.00	0.38*	0.55

**Figure 1:**
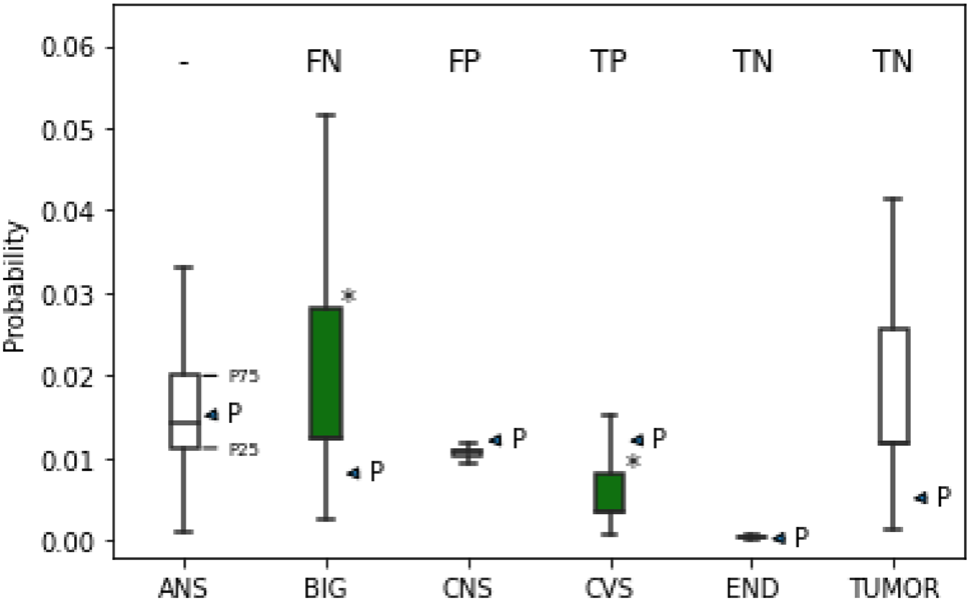
Model evaluation on HAD type prediction.

Figure 1 shows an example of the evaluation process in the HAD type classification cycle. The patient in this visit was prescribed with CVS and BIG (indicated in the green/opaque drug prescription box plot). Probabilities at P25 and P75 (lower and upper side of the box) represent the cut points to separate predicted non-HAD (below P25) from HAD (above P75).

According to the cut point of HAD use, CVS and CNS were recommended for prescription in this case (probability above P75). CVS was correctly predicted and counted as true positive (TP) whereas the probability of CNS was incorrectly predicted and counted as false positive (FP). The prediction of non-HAD use was not interpreted because we did not consider the prediction of non-HAD in the HAD type classification cycle. The probability of BIG was below P25 and misclassified, resulting in a false negative (FN). The probability of Tumor and END were below P25 and correctly recommended no HAD use, resulting in a true negative (TN). The probability of ANS was in between 25th percentile and 75th percentile which was not interpreted and excluded from the analysis. The results of TP, FP, TN, and FN were used to calculate accuracy, precision, recall, and F1-score.

#### Post-hoc result readjustment

In this cycle, we did not readjust the cut points like the HAD binary classification cycle. Therefore, we skipped the evaluation process on an evaluation set and only applied HAD percent and ICD10 percent to manually evaluate the results.

##### HAD percent

Similar to the definition of HAD percent in HAD binary classification cycle, the percentages of HAD use were calculated from the prescription and ICD10 data. In this cycle, the HAD percent was separately calculated into HAD types in ANS, BIG, CNS, CVS, END, and Tumor.

##### ICD10 percent

To find the type of HAD that was most commonly prescribed for certain ICD10s, we designed another indicator to interpret and evaluate the prediction results called “ICD10 percent”. ICD10 percent represents the percentage of visits that have a specific type of HAD prescribed out of all visits with the same specific ICD10 code. For instance, to evaluate the BIG type of HAD prescription, the percentage of ICD10 for Z921 (Personal history of long term(current) use of anticoagulants) was 94.11%, which mean out of 100 patients with ICD10 code “Z921”, there were approximately 94 visits that showed a history of BIG-type HAD prescription in the records. Based on this information, we used ICD10 percent to clinically validate the appropriateness of HAD usage for a specific ICD10 code.

#### HAD–ICD10 mismatch

Overall, we considered HAD prescriptions with model prediction and readjustment process to detect the inconsistent use (outlier) of HAD which could be interpreted as an incidence of prescription error. Therefore, we aim that our HAD screening approach is capable of searching for and finding visits with HAD–ICD10 mismatches. Then, we discussed the incidence of HAD–ICD10 mismatches as an unrelated use of HAD, dividing the appropriate use of HAD into two groups. First, the HAD group for symptomatic treatment related to presenting symptoms or signs but not specifically related to ICD10s. For example, patients presenting with nose blockage or running nose were prescribed with Pseudoephedrine (nasal decongestant). Patients with insomnia were prescribed with Alprazolam (anti-anxiety disorder). An example of HAD–ICD10 mismatch in this case is when the patients with I10 (Essential hypertension) were prescribed with Pseudoephedrine. Most patients with I10 (Essential hypertension) were typically treated with anti-hypertensive drugs whereas Pseudoephedrine has medically no role in Essential hypertension treatment (according to the hypertension guidelines 2020^30^). The prescription of Pseudoephedrine is interpreted as an error. Second, the HAD group for definite treatment such as anticoagulant, cytotoxic agent and antiarrhythmic drug. These HAD groups are obligated to follow a standard indication because the misuse of those drugs may lead to fatal or severe side effects. Any of those HAD uses apart from the standard guidelines were classified as HAD–ICD10 mismatch. Therefore, local guidelines, expert agreements, or controversy recommendations were discussed as a mismatch. For example, patients with long term essential hypertension with complications such as coronary artery diseases, stroke, chronic kidney disease, heart failure or chronic obstructive pulmonary disease were prescribed drugs relating to the guidelines on each disease and/or its complications^31–34^. However, we found that some patients in this group were prescribed with Maforan. Maforan is an anticoagulant drug which is commonly used for preventing thromboembolism in abnormalities related to abnormal blood flow such as valvular heart diseases^35^ and deep vein thrombosis^36^. According to clinical standard guidelines of anticoagulant use, the prescription of Maforan was not the defined treatment for any of those diseases or complications although it was sometime used and accepted according to local guidelines. This result was interpreted as HAD–ICD10 mismatch. In the next section, we demonstrate the results of applying this HAD screening protocol and measure the efficiency of the protocol to detect HAD prescription errors.

### Ethics approval and consent to participate

The study was carried out in accordance with guidelines of Faculty of Medicine, Chiang Mai University (Study code: PHY-2562-06160). All methods were carried out with exemption criteria (waiver of informed consent) and the need for informed consent was waived by the Research Ethics Committee of Faculty of Medicine, Chiang Mai University in accordance with the Faculty of Medicine, Chiang Mai University Ethics regulations.

## Results

### Data set

The 168,145 visits of the OPD data set (991,270 prescriptions) were split (sampling without replacement) to 35,268 (200,000 prescriptions) and 132,877 (791,270 prescriptions) visits for HAD binary classification cycle and HAD type classification cycle, respectively. The 11,167 visits of the IPD data set (1,200,000 prescriptions) were split (sampling without replacement) to 3613 (400,000 prescriptions) and 7554 (800,000 prescriptions) visits for HAD binary classification cycle and HAD type classification cycle, respectively. Tables 5 and 6 shows the distribution of HAD types on age and gender in the OPD and IPD data sets. The average age of visits with HAD use ranges between 40s and 50s year-old in both groups. The most common HAD type was BIG in OPD data set and CNS in IPD data set. The data sets of the HAD binary classification cycle were directly passed to the model training and validation process. Each data set of the HAD binary classification cycle (35,268 OPD and 3613 IPD visits) was shuffled with a constant seed and split to training and test sets using a fixed-split technique with 75:25 ratio. In the HAD type classification cycle, the data sets were additionally filtered with the cut points (0.06 for OPD and 0.30 for IPD data sets) and excluded visits below the cut points before passing the data to the model training and validation process. The final OPD and IPD visits of the HAD type classification cycle were mapped with actual HAD types resulting in 43,513 (39,162 visits) and 10,690 (8062 visits) instances respectively. The average number of drug types per visit were 1.11 in the OPD and 1.33 in the IPD data sets. Then, the data sets were split into training and test sets using the same protocol as the HAD binary classification cycle. Tables 7 and 8 show the result of data split and the percentage of HAD presented in a bracket in correspondence with the data set. The percentage of HAD in the OPD data increased two times after applying the cut point while the percentage of HAD in the IPD data set remained in the same proportion.

**Table 5:** HAD type distribution in OPD data set.

HAD types	Age			Gender	
Cycle 1	Count	Mean	Std	Male	Female
ANS	1700	39.82	12.46	602	1,098
BIG	2538	55.67	10.34	1227	1311
CVS	371	51.52	13.40	145	226
CNS	734	44.01	17.75	398	336
END	45	53.93	15.61	34	11
Tumor	1023	47.43	13.19	256	767
Non-HAD	193,589	47.82	14.70	77,906	115,683
Total	200,000	–	–	80,568	119,432
Cycle 2	Count	Mean	Std	Male	Female
ANS	4011	39.88	13.34	1390	2621
BIG	10,628	57.56	11.91	4933	5695
CVS	1473	54.05	12.62	636	837
CNS	2717	45.82	20.86	1456	1261
END	160	55.73	11.77	100	60
Tumor	5177	49.13	11.77	1421	3756
Non-HAD	767,104	49.05	15.84	307,749	459,355
Total	791,270	–	–	317,685	473,585
Grand total	991,270	–	–	398,253	593,017

**Table 6:** HAD type distribution in IPD data set.

HAD types	Age			Gender	
Cycle 1	Count	Mean	Std	Male	Female
ANS	3673	54.63	26.47	2435	1238
BIG	3170	60.15	19.73	1525	1645
CVS	7339	52.63	24.00	3964	3375
CNS	9602	50.00	25.22	5147	4455
END	2066	50.90	24.82	1146	920
Tumor	5321	47.53	20.00	2638	2683
Non-HAD	368,829	55.50	23.07	194,439	174,390
Total	400,000	–	–	211,294	188,706
Cycle 2	Count	Mean	Std	Male	Female
ANS	7016	53.50	27.13	3668	3348
BIG	6335	58.72	20.67	2900	3435
CVS	12,942	51.92	25.26	6625	6317
CNS	20,032	47.96	26.10	10,204	9828
END	3875	47.05	27.04	2104	1771
Tumor	10,200	46.55	21.25	5464	4736
Non-HAD	739,600	53.56	53.56	377,839	361,761
Total	800,000	–	–	408,804	391,196
Grand total	1,200,000	–	–	620,098	579,902

**Table 7:** Distribution HAD visits in OPD data set.

Cycle 1	N (HAD%)	Cycle 2	N (HAD%)
Training set	26,451 (8%)	Training set	27,977 (16%)
Test set	8817 (9%)	Test set	11,185 (18%)
Total	35,268 (8%)	Total	39,162 (16%)

**Table 8:** Distribution HAD visits in IPD data set.

Cycle 1	N (HAD%)	Cycle 2	N (HAD%)
Training set	2710 (61%)	Training set	4900 (63%)
Test set	903 (62%)	Test set	3162 (57%)
Total	3613 (61%)	Total	8062 (61%)

### Machine learning algorithm selection

The baseline accuracy values of DummyClassifier were 0.84 and 0.52 internally validated on the OPD and IPD data sets with HAD and non-HAD labels. The performances of all tested classifiers were better than the baseline in both data sets. The Gradient boosting classifier provided the best performance among the other classifiers with 0.93 and 0.77 accuracy on the OPD and IPD data sets. We applied Grid search to optimize the max_depth parameter trading off between model efficacy and simplicity. As a result, the best max_depth was set to 1 with 0.93 and 0.75 accuracy for the OPD and IPD data sets. The Gradient boosting classifier with max_depth=1 was selected to develop the drug verification protocol in both cycles.

### Relevant features

**Figure 2:**
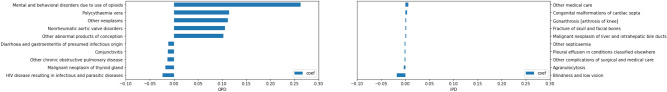
HAD binary classification: Feature importance in OPD and IPD data sets.

**Figure 3:**
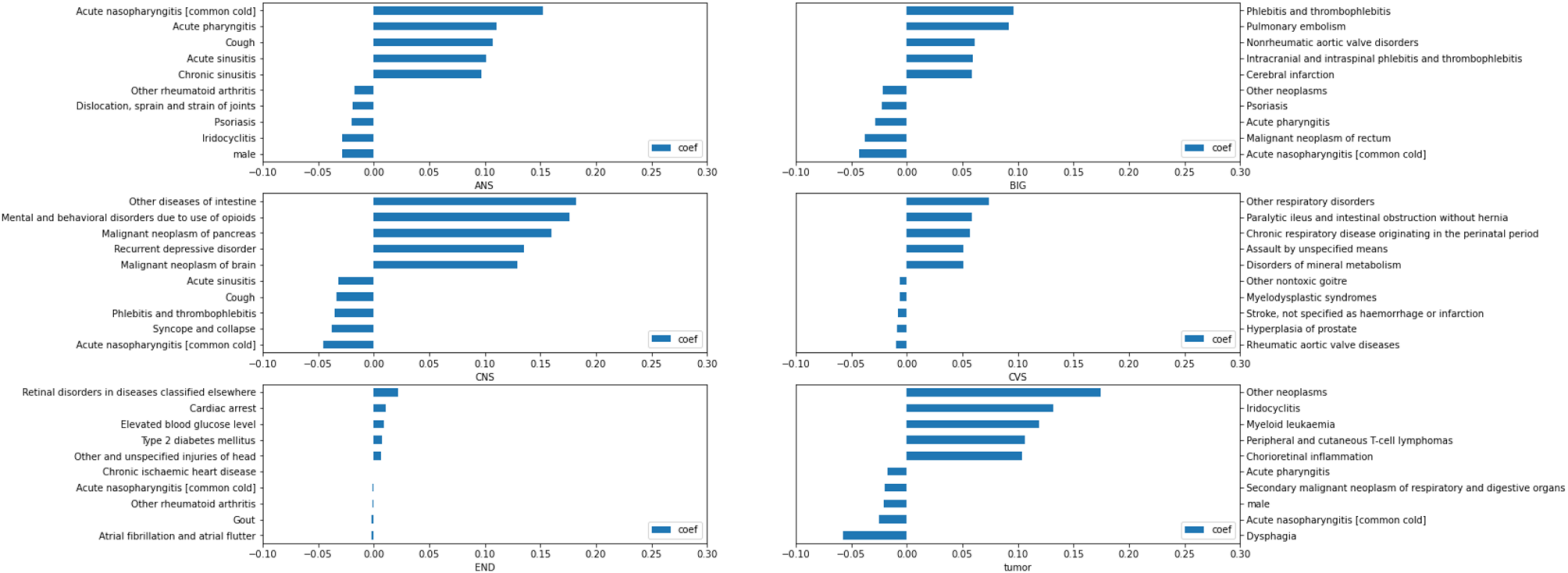
HAD type classification: Feature importance in OPD data set.

**Figure 4:**
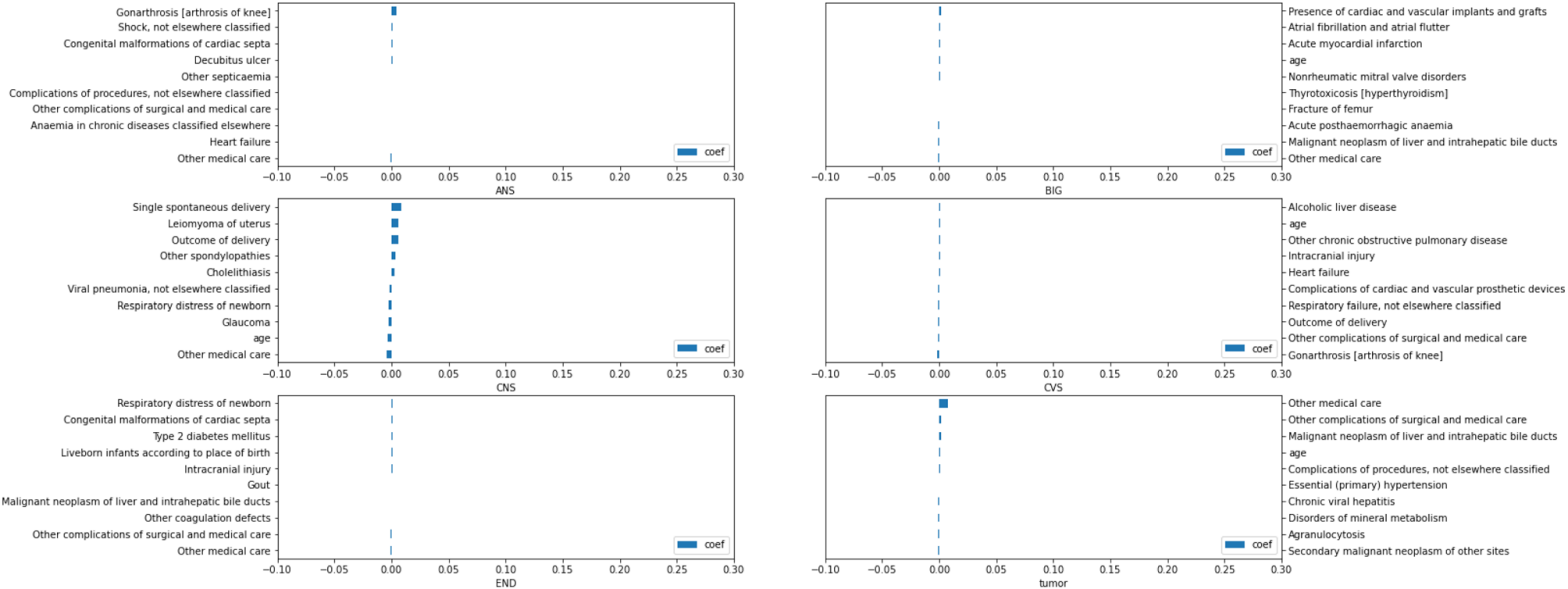
HAD type classification: Feature importance in IPD data set.

Figures 2, 3, and 4 show the top 5 most positive (label 1–5 on the y axis) and negative (label 6–10 on the y axis) relevant features that contributed to the model prediction of the HAD binary and type classification. Generally, the coefficient numbers in OPD data sets were higher than IPD data sets in both cycles.

### Cycle 1: HAD binary classification cycle

#### HAD binary classification

**Figure 5:**
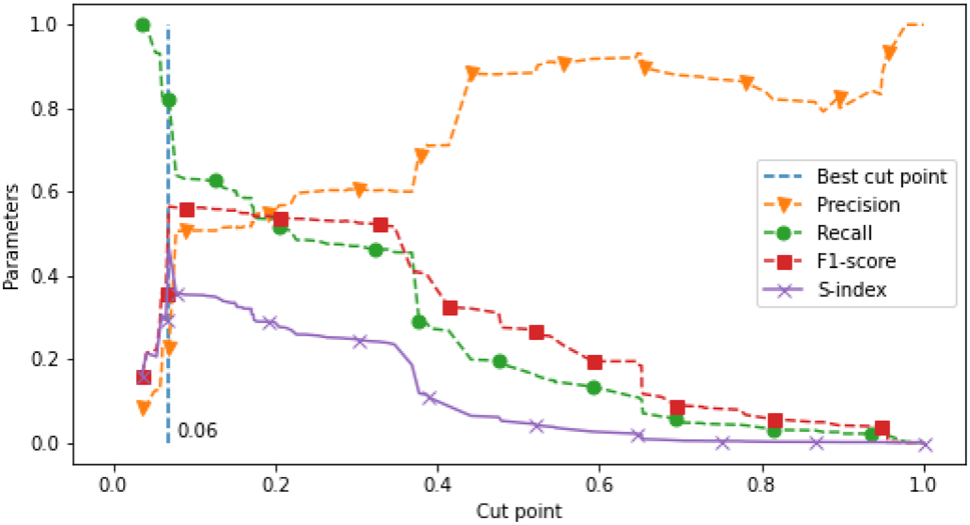
HAD cut point selection in OPD data set.

**Figure 6:**
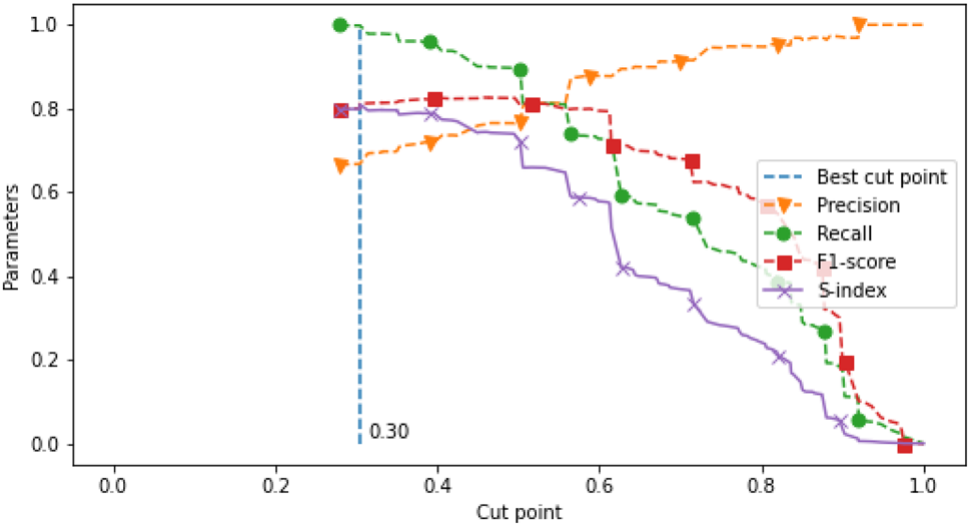
HAD cut point selection in IPD data set.

Starting with the cut point selection process, Figs. 5 and 6 show the plot between the evaluation metrics (y-axis): precision (orange dash line with a triangle mark); recall (green dash line with a circle mark); F1-score (red dash line with a square mark); and S-index (purple solid line with a cross mark) and the moving cut points (x-axis). The vertical dash line draws through the selected cut points at 0.06 and 0.30 where the value of S-index is the highest for the OPD and IPD data sets, respectively. Predictions above the cut points were interpreted as HAD use, otherwise, non-HAD use.

The selected cut points were used to classify HAD use. Tables 9 and 10 show the model performance of HAD binary classification internally validated with OPD (n = 8817) and IPD (n = 903) test sets. As a result of maximizing the S-index, we determined the models with high recall and low precision for predicting the HAD class.

**Table 9:** Prediction performance of HAD binary classification in OPD data set.

HAD type	Accuracy	Precision	Recall	F1-score	N
Non-HAD	0.75	0.98	0.74	0.84	8,063
HAD	0.75	0.23	0.83	0.36	754

**Table 10:** Prediction performance of HAD binary classification in IPD data set.

HAD type	Accuracy	Precision	Recall	F1-score	N
Non-HAD	0.69	0.98	0.18	0.30	341
HAD	0.69	0.67	1.00	0.80	562

#### Post-hoc result readjustment

Table 11 shows the top five highest HAD percent and the corresponding specific false negative ratio (sFNR) in OPD and IPD data sets. The results show that ICD10s with high HAD percent relate to low sFNR.

**Table 11:** Top 5 the highest HAD percent with specific false negative ratio (sFNR).

ICD10	HAD %	sFNR
**Outpatient data set**		
Z921 History of long term use of anticoagulants	96.03	0.00
I050 Mitral stenosis	92.92	0.00
Z952 Presence of prosthetic heart valve	92.02	0.16
I071 Tricuspid insufficiency	84.09	0.05
I489 Atrial fibrillation, unspecified	79.03	0.06
**IPD data set**		
Z511 Chemotherapy session for neoplasm	98.42	0.00
J960 Acute respiratory failure	97.29	0.00
E834 Disorders of magnesium metabolism	96.96	0.00
I48 Atrial fibrillation	94.17	0.00
C910 Acute lymphoblastic leukaemia	92.30	0.00

**Figure 7:**
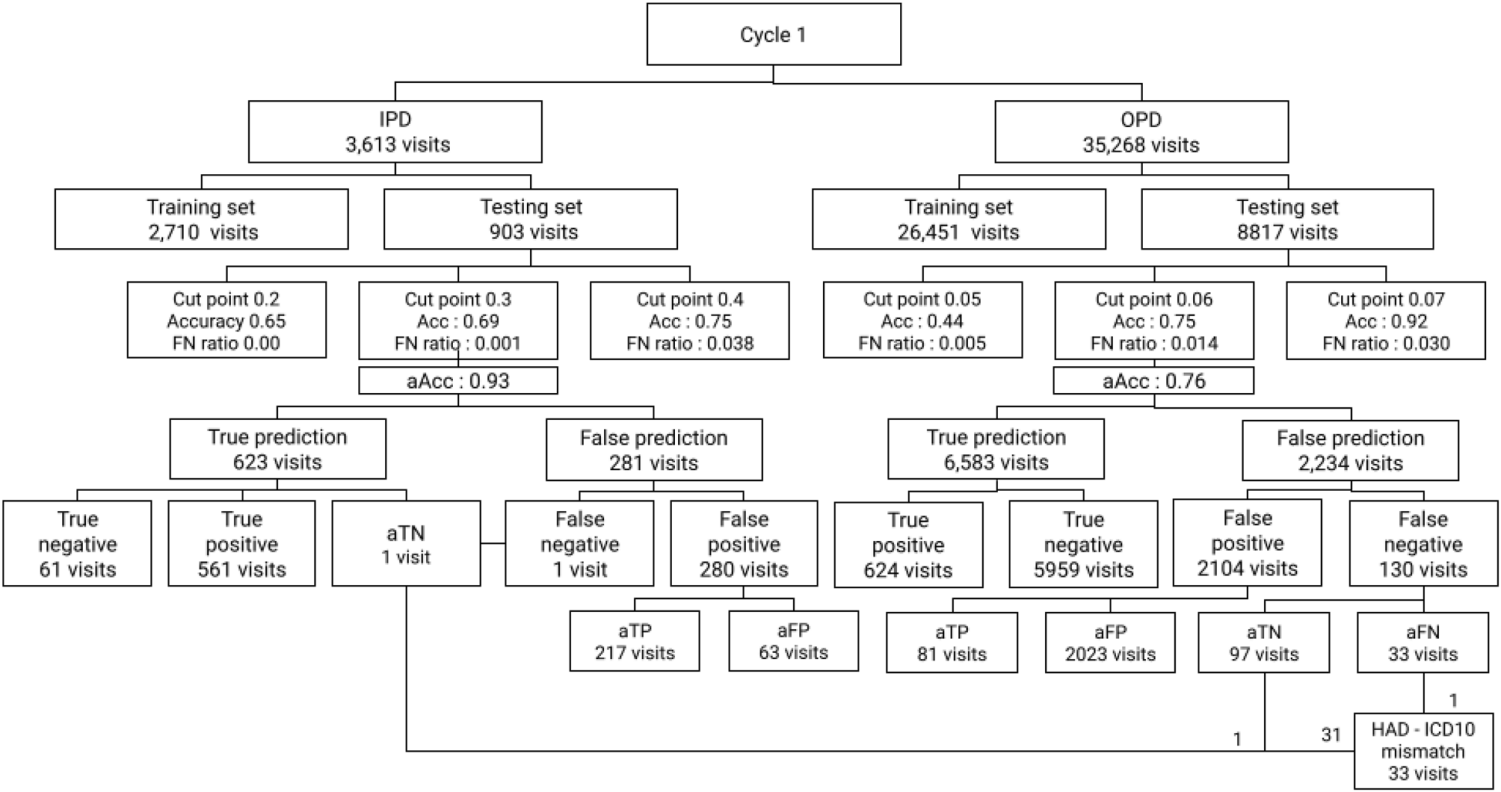
HAD classification result in Cycle 1.

False positive, false negative, true positive and true negative were adjusted by HAD percent. Figure 7 shows the results of adjustment. We found 1 false negative in the IPD data set and 130 false negatives in the outpatient data set. After further investigation, the false negative in the IPD data set was readjusted to be a true negative, resulting in no false negatives remaining from the analysis. The 130 false negatives in the OPD data set were readjusted to 97 true negatives (aTN). For those 130 false negatives who received HAD even with low HAD percent and prediction, 65 cases were manually reviewed to determine that HAD were appropriately prescribed whereas the ICD10s in a further 32 cases were not associated with HAD according to the standard clinical guidelines. In contrast, one visit in the adjusted false negative (aFN) was highly associated with HAD because of high HAD percent but the model incorrectly predicted low probability of HAD use.

#### HAD–ICD10 mismatch

Regarding the readjustment results in Fig. 7, one HAD–ICD10 mismatch was found in the IPD data set and 32 mismatches were found in the OPD data set. We further analyzed the mismatches at the drug name level. Table 12 shows the types of HAD ranked by frequency of mismatch (presented in the proportion column) in the outpatient (Out) and IPD data sets. The top two mismatched HAD are Pseudoephedrine and Alprazolam (63.6% and 21.2% respectively). The most common ICD10 with the highest frequency of HAD–ICD10 mismatch is essential (primary) hypertension (18 visits), which were mostly prescribed with Pseudoephedrine.

**Table 12:** HAD–ICD10 mismatch.

	OPD	IPD	Proportion (%)
**Mismatch validation in cycle 1**			
Pseudoephedrine F/C tab 60 mg	20	1	63.6
Alprazolam tab 0.25 mg	7	0	21.2
Maforan tab 1,2,3,5 mg	2	0	6.1
Heparin inj 5000 u/ml 5 ml	1	0	3.0
Emthexate tab 2.5 mg	1	0	3.0
Methadone sol (1 mg/ml) 10 ml	1	0	3.0
Total	32	1	100.0
**Mismatch evaluation in cycle 1**			
Maforan tab 1,2,3,5 mg	32	2	29.1
Alprazolam tab 0.25 mg	18	1	16.2
Emthexate tab 2.5 mg	18	1	16.2
Pseudoephedrine F/C tab 60 mg (ANS)	17	3	17.1
Cycloxan tab 5 mg	6	0	5.1
Hydrea cap 500 mg	5	0	4.3
Endoxan inj 1,200 mg	2	0	1.7
Kemocarb inj	2	0	1.7
Intaxel inj 300 mg/50ml	1	0	0.9
Fentanyl 50 mcg/hr	1	0	0.9
Midazolam inj 5 mg/ml	1	0	0.9
Morphine tab 10 mg	1	0	0.9
Vincristine inj 1mg/ml	1	0	0.9
Heparin inj 5000 u/ml 5 ml	0	1	0.9
Zolpidem tab 10 mg	0	1	0.9
Magnesium sulfate (CVS)	0	3	2.6
Total	105	12	100.0
**Mismatch validation in cycle 2**			
Pseudoephedrine F/C tab 60 mg (ANS)	17	0	58.6
Alprazolam tab 0.25 mg (CNS)	2	0	6.9
Maforan tab 2,3,5 mg (BIG)	4	0	13.7
Methrotexate (tumor)	5	0	17.2
Magnesium sulfate (CVS)	1	0	3.45
Total	29	0	100.0

### Cycle 2: HAD type classification cycle

#### HAD inclusion

In this cycle we used the trained machine learning models from the HAD binary classification cycle to predict the probability of 132,877 OPD (containing 9382 visits with HAD prescriptions) and 7554 IPD (containing 4585 visits with HAD prescriptions) visits. Visits with the probability below or equal 0.06 for OPD data set and 0.30 for IPD data set were excluded for analysis in this cycle. Table 13 shows the number distribution of HAD types which were excluded and included from the data sets. The remaining data sets were transformed to map with HAD labels and split into training and test sets as presented in Tables 7 and 8.

**Table 13:** Number of excluded and excluded HAD prescriptions in OPD and IPD data sets.

HAD types	OPD HADs (142,137)		IPD HADs (14,812)	
	Excluded	Included	Excluded	Included
ANS	633	2583	5	589
BIG	381	2294	3	673
CVS	20	620	1	1677
CNS	572	763	3	2577
END	29	27	0	496
Tumor	327	1744	1	1249
Non-HAD	93,646	38,498	584	6954
Total	95,608	46,529	597	14,215
Total visits	94,006	39,162	584	8062

#### Model evaluation of HAD binary classification

**Figure 8:**
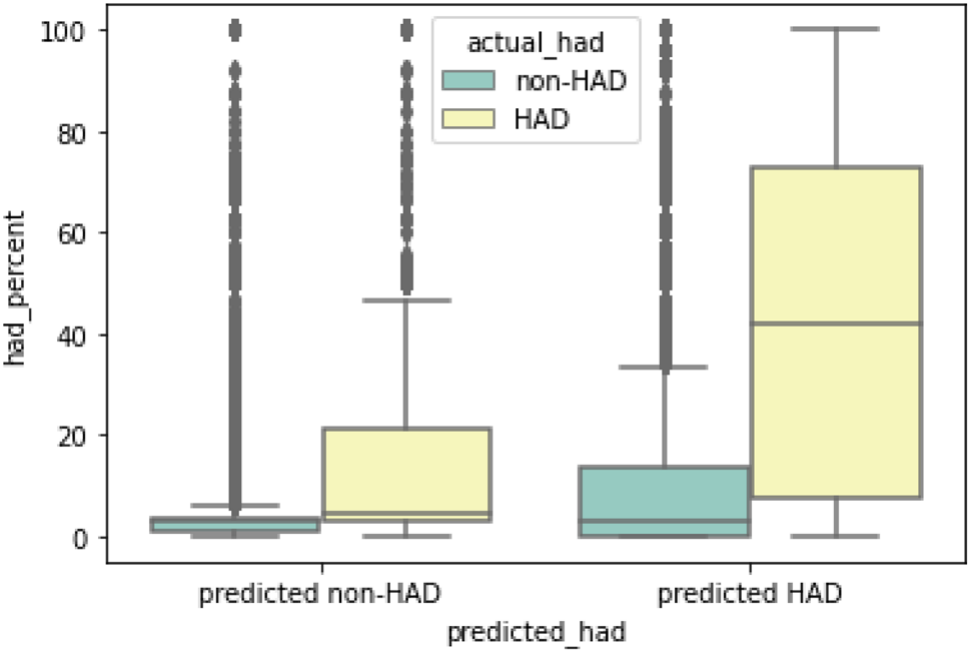
HAD binary classification result in OPD data set.

**Figure 9:**
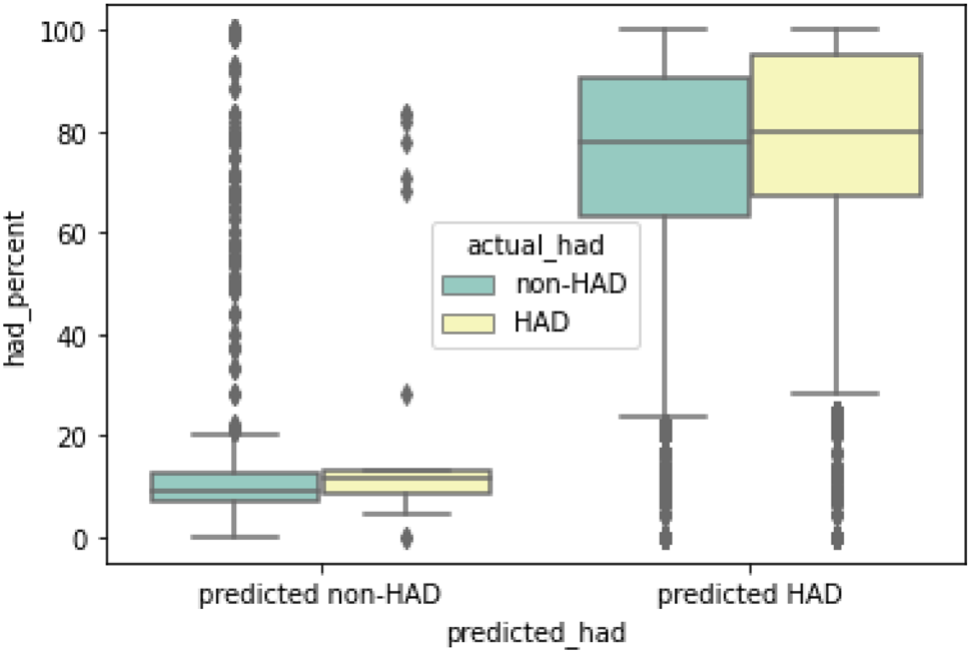
HAD binary classification result in IPD data set.

By applying the cut points, the 94,006 OPD visits containing 1946 visits with actual HAD (false negative, FN) and 584 IPD visits containing 13 visits with actual HAD (false negative, FN) were excluded from the data sets and passed to reevaluate with the same process as for the HAD binary classification cycle. The FN visits were reevaluated with HAD percent. Figures 8 and 9 shows the reevaluation results of HAD binary classification in OPD and IPD dataset comparing the distribution of HAD percent between actual HAD and non-HAD visits. Visits with high HAD percent were mostly present in predicted HAD group of OPD data set while there is no difference of HAD percent between non-HAD and HAD groups of IPD data set. From 1946 FN visits, 1462 OPD and 10 IPD visits with actual HAD and HAD percent lower than 50 were manually evaluated for HAD–ICD10 mismatches. On the other hand, 278 OPD and 3 IPD visits with actual HAD and HAD percent equal or greater than 50 were manually reviewed for model misclassification.

#### HAD type classification

**Figure 10:**
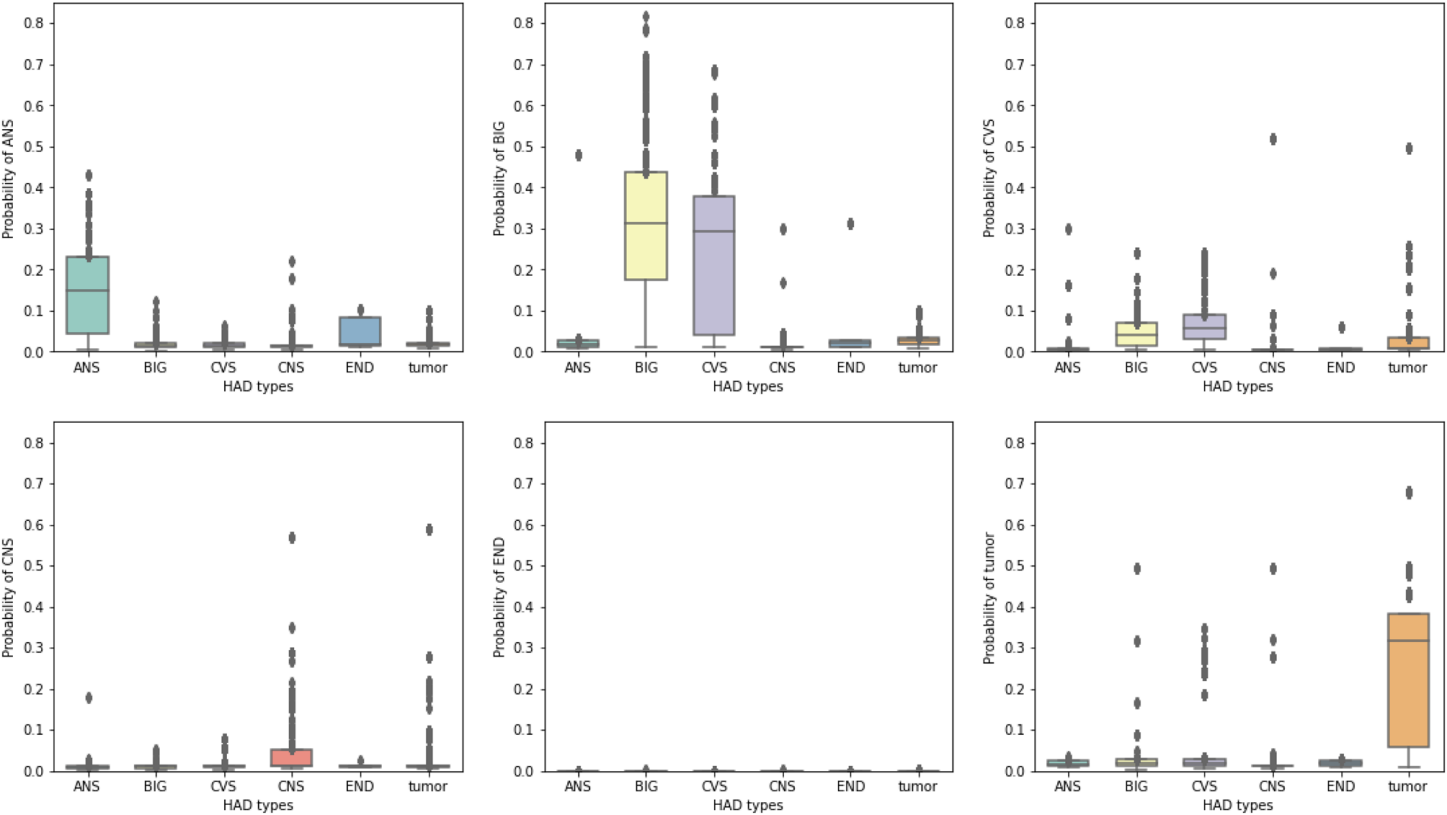
HAD type classification in OPD data set.

**Figure 11:**
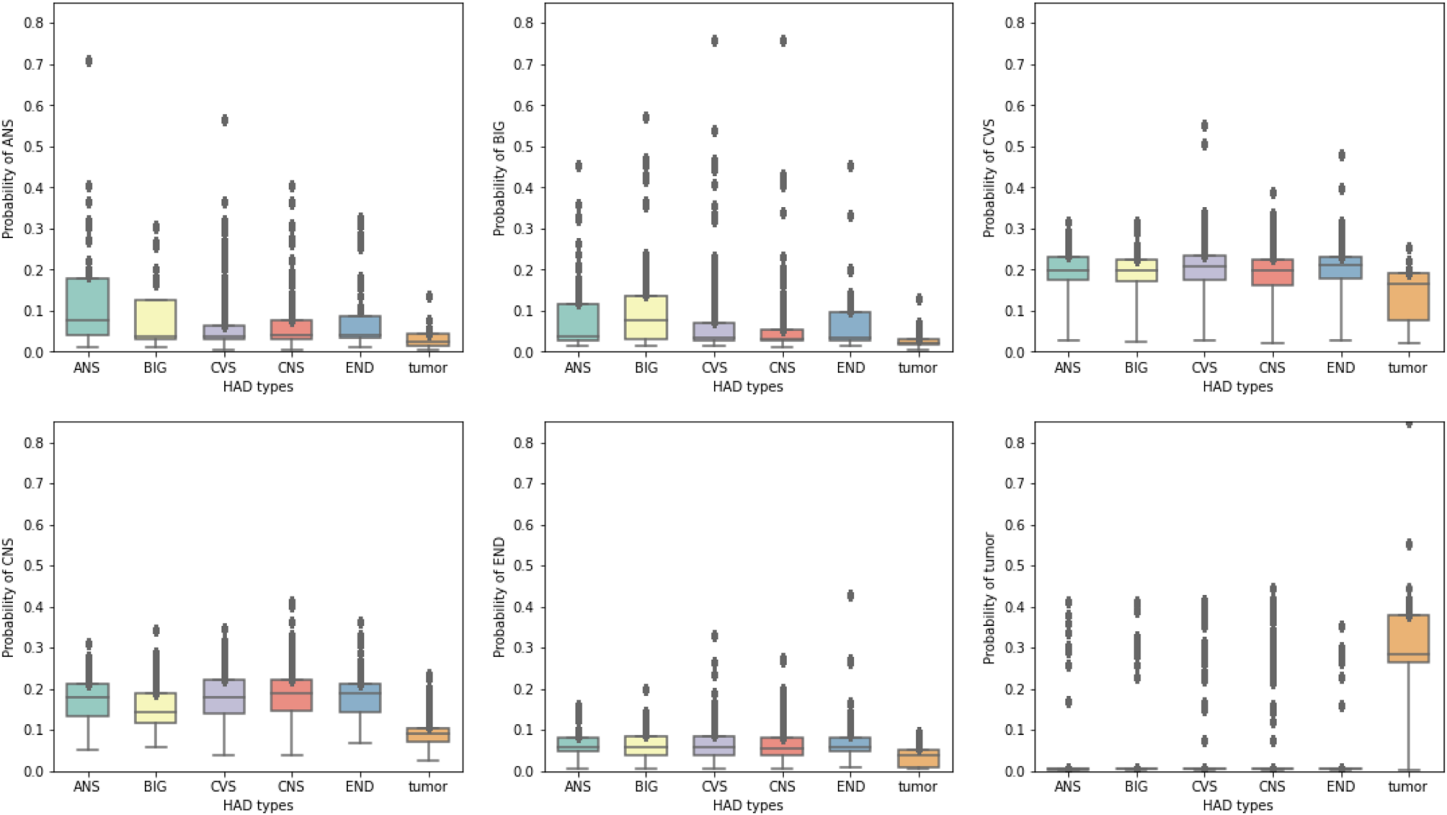
HAD type classification in IPD data set.

The P25 and P75 cut points were used to classify the HAD type tested again an actual label, then we interpreted the results to TP, TN, FP, and FN and used these values to calculate the evaluation metrics. Table 14 shows the prediction performance by standard metrics including accuracy, precision, recall, and F-1 score of each different HAD type and the average in the OPD data set indicated in a value range from 0 to 1. The model performed with very high recall and low precision across all HAD types and predicted BIG with the best accuracy. Table 15 shows the prediction performance by standard metrics including accuracy, precision, recall, and F-1 score of each different HAD type and the average in the IPD data set indicated in a value range from 0 to 1. Similar to the model performance in the OPD data set, the model performed with very high recall and low precision across all HAD types while the best accuracy was present in Tumor type. Figures 10 and 11 show the distribution of HAD type classification in OPD and IPD data sets. The HAD type classification in OPD data set shows a strong prediction pattern of single (ANS, tumor) and double (BIG & CVS) HAD use while HAD type classification in IPD data set shows no significant pattern of HAD use except in Tumor type.

**Table 14:** Prediction performance on each HAD type in OPD data set.

HAD type	Accuracy	Precision	Recall	F1-score	N
ANS	0.61	0.20	0.96	0.33	649
BIG	0.68	0.18	0.94	0.30	590
CVS	0.63	0.04	0.94	0.08	147
CNS	0.60	0.04	0.95	0.07	199
END	0.63	0.00	0.43	0.00	8
Tumor	0.65	0.12	0.88	0.21	441
Avg	0.64	0.12	0.93	0.20	405

**Table 15:** Prediction performance on each HAD type in IPD data set.

HAD type	Accuracy	Precision	Recall	F1-score	N
ANS	0.55	0.11	0.95	0.20	142
BIG	0.57	0.14	0.96	0.25	166
CVS	0.60	0.21	0.90	0.34	417
CNS	0.61	0.28	0.80	0.42	641
END	0.56	0.12	1.00	0.21	134
Tumor	0.66	0.33	0.98	0.50	308
Avg	0.59	0.20	0.93	0.32	301

#### Post-hoc result readjustment

Tables 16 and 17 show the three highest ICD10 percentages for each HAD type in OPD and IPD data sets. In outpatients, ANS was commonly used for diseases related to upper respiratory infection, CNS for pain control in injuries or tumors, BIG for cardiovascular diseases, Tumor for tumors, and CVS for cardiovascular diseases and tumors. The pattern of HAD use in the IPD group was found in similar disease groups to the outpatient results but the diseases were more severe.

**Table 16:** The three highest numbers of ICD10 percent for each HAD type in OPD data set.

Type	ICD10	ICD10 %
ANS	J019 Acute sinusitis	62.29
	J00 Acute nasopharyngitis	52.43
	J069 Acute upper respiratory infection	40.81
CNS	S430 Dislocation of shoulder joint	58.20
	C795 Secondary malignant neoplasm of bone	34.28
	C679 Malignant neoplasm of bladder	23.52
BIG	Z921 History of long term use of anticoagulants	94.11
	I050 Mitral stenosis	93.68
	Z952 Presence of prosthetic heart valve	93.08
Tumor	C833 Diffuse large B-cell lymphoma	100.00
	C50.9 Malignant neoplasm of breast	92.50
	Z511 Chemotherapy session for neoplasm	89.67
CVS	C539 Malignant neoplasm of cervix uteri	29.60
	I420 Dilated cardiomyopathy	21.97
	Z511 Chemotherapy session for neoplasm	19.57

**Table 17:** The three highest numbers of ICD10 percent for each HAD type in IPD data set.

Type	ICD10	ICD10 %
ANS	R572 Septic shock	94.36
	J960 Acute respiratory failure	65.85
	N179 Acute renal failure	56.00
CNS	D62 Acute posthaemorrhagic anemia	82.38
	J960 Acute respiratory failure	77.00
	E871 Hyposmolality and hyponatraemia	59.10
BIG	I215 Atherosclerotic heart disease	67.64
	I48 Atrial fibrillation	65.82
	I489 Atrial fibrillation, unspecified	69.56
Tumor	Z511 Chemotherapy session for neoplasm	97.31
	C910 Acute lymphoblastic leukaemia	90.90
	D70 Agranulocytosis	66.66
END	E119 Type 2 diabetes	30.00
	D62 Acute posthaemorrhagic anemia	27.67
	I215 Atherosclerotic heart disease	25.73
CVS	E834 Disorders of magnesium metabolism	92.00
	E876 Hypokalaemia	72.55
	E871 Hyposmolality and hyponatremia	66.00

**Figure 12:**
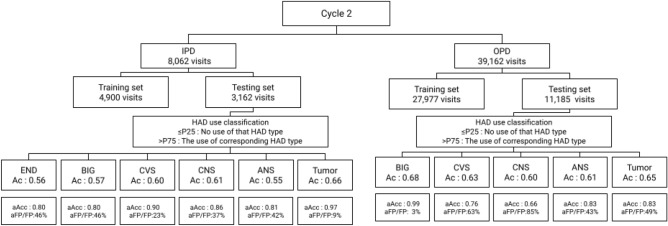
HAD classification result in Cycle 2.

Similar to the HAD binary classification cycle, false positive, false negative, true positive and true negative were adjusted by HAD percent. Figure 12 shows the results of HAD type prediction. After adjusting prediction results with HAD percent, the accuracy improves across all HAD types.

#### HAD–ICD10 mismatch

**Figure 13:**
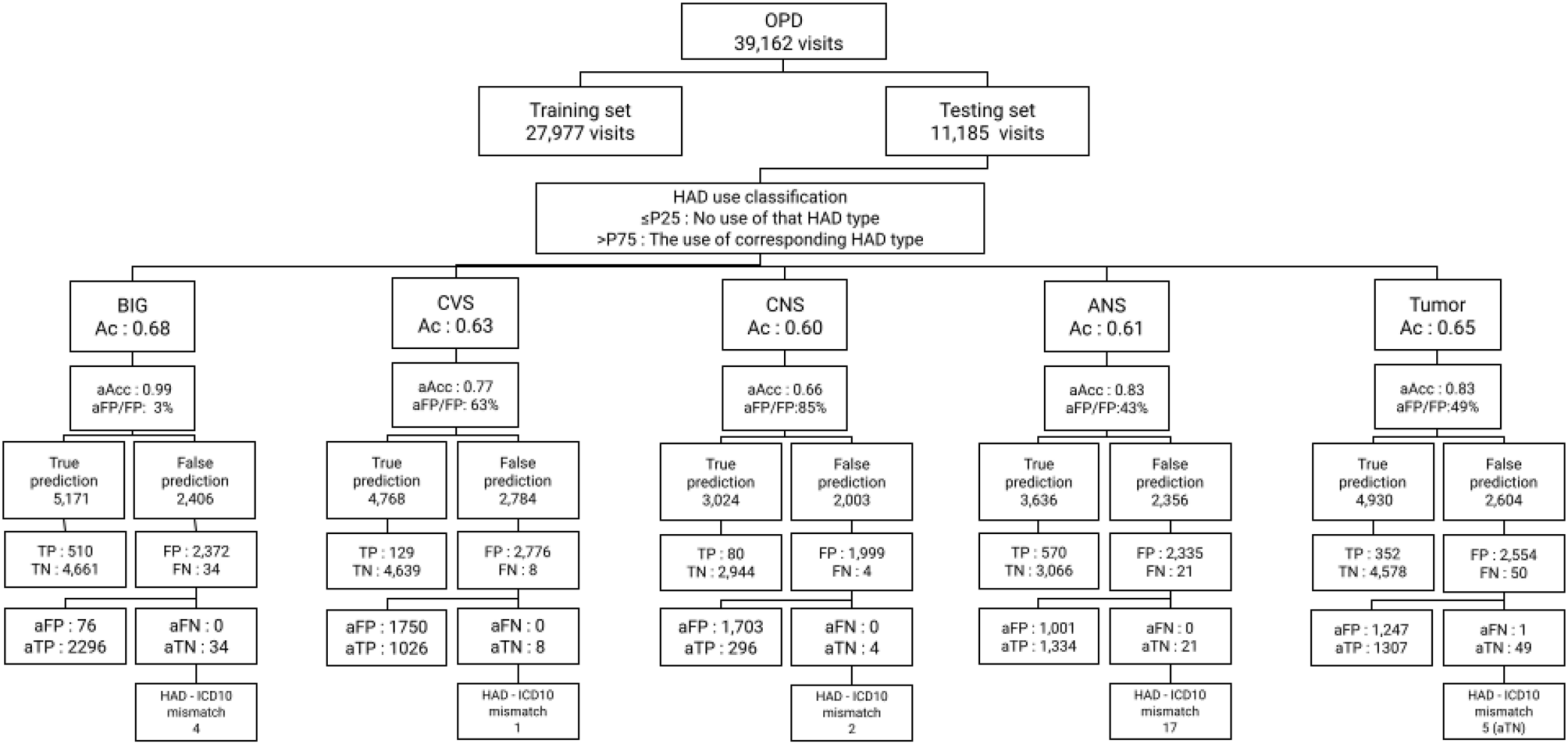
HAD–ICD10 mismatch in outpatient data set in Cycle 1.

**Figure 14:**
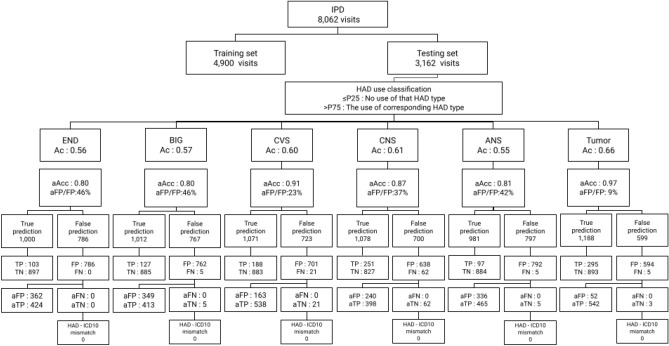
HAD–ICD10 mismatch in IPD data set in Cycle 2.

After adjusting the prediction result, there were no HAD–ICD10 mismatches found in the IPD data set (see Fig. 14). For the outpatient data set, we found 29 HAD–ICD10 mismatches (see Fig. 13). The highest number of HAD–ICD10 mismatches were present in ANS (17 cases) which refers to Pseudoephedrine as found in the result of the eHAD type classification cycle (see the full results of HAD–ICD10 mismatch for each HAD type in Fig. 13). There was one adjusted false negative (aFN) in Tumor. However, the one aFN in Tumor was not classified to the HAD–ICD10 mismatch group after being reviewed according to the standard guidelines^37^.

## Discussion

### Cycle 1: HAD binary classification cycle

#### Selection of cut point

The development of HAD verification is a challenging task. In this study, we intentionally developed an approach to screen the appropriateness of HAD use at the Maharaj Nakorn Chiang Mai hospital. Although the number of inappropriate use of HAD incidence is typically low, even just one HAD misuse may cause serious consequences when it is undetected. Because of the low incidence, we set the criteria to measure the prediction performance with an emphasis towards detecting faulty prescriptions. Therefore, we weighted the model prediction preference towards high recall. However, 100% recall might not provide us the best model because of too many false positives. We thus considered adding F-1 score to measure the balance of prediction performance resulting in using S-index (see Eq. 1).

#### Result adjustment

We used HAD percent to adjust the number of true positive, true negative, false positive, and false negative. These parameters indicated the corrected result based on statistical HAD use which widened the perspective of the appropriateness of HAD prescriptions regardless of the incidence of HAD use. For the outpatient data set, at cut point 0.06, we found 2023 out of 2104 (FP) visits was interpreted to adjusted false positive (aFP). The cause of this high prediction value of false positives was the pattern of multiple drug use in the visits (discussed later in the EAMU section). In the false negative group, we found only 33 adjusted false negatives (aFN) from a total of 130 false negatives (FN) which means only one fourth of total false negatives predicted by the machine learning model are actually false. Within the 33 aFN, we found one aFN which was a HAD–ICD10 mismatch and interpreted as a prediction error. On the other hand, we found 31 visits out of 97 adjusted true negatives (aTN) actually received HAD in contrast with the evidence of HAD prediction, HAD percent, and clinical guidelines, which were explored in the HAD–ICD10 mismatch section. The IPD data set, at cut point 0.3, found 280 false positives out of 281 false predictions. Similar to the pattern of false predictions found in the OPD data set, the false prediction visits contained ICD10s with low HAD association such as brachial plexus disorder (a disease of peripheral nerve injury at the level of plexus running in shoulder), chronic venous insufficiency (a disorder of structural or functional of vein leading to localized blood congestion), and hydrocele (swelling in the scrotum). The three ICD10s showed low HAD percent (33%) and low individual prediction of HAD use (0.50, 0.42 and 0.39 respectively). However, the model predicted high probability of HAD use in this case opposed to the HAD percent because the treatment of those ICD10s alone were not directly related to HAD use but they were often diagnosed along with other ICD10s which were highly related to HAD use. Because of this coincident pattern, the model was misled to learn the incorrect relationship and calculated a high prediction value for those ICD10s. After correcting the prediction results with HAD percent, the false positives were reduced to 63 (aFP) and the accuracy increased to 0.93. For the remaining false predictions, we found one false negative which was readjusted to true negative (aTN) by HAD percent. The result of manual evaluation included this event in the HAD–ICD10 mismatches. However, HAD percent is not a perfect parameter to determine the HAD use. In the case of too small a number of visits, the same sample size leads to low reliability. For example, we could not say that HAD is totally associated with an ICD10 when it is only used once in the entire data set. Also, the complex relationship between ICD10s when there are multiple ICD10s within the same visit limits the interpretation of HAD percent to the use of HAD.

#### Prediction performance: OPD

The initial cut point selection of probability in the OPD data set was at 0.06. The model performance on HAD prediction at this cut point showed accuracy, precision, recall and F1-score were 0.75, 0.23, 0.83 and 0.36, respectively. Although the precision score indicating the ability to predict HAD prescription parameter is quite low, the remaining standard parameters present a good HAD prediction performance, especially in the top 5 of the HAD percent table (see Table 11). The performance of the model prediction was capable of detecting the pattern of HAD use because those ICD10s contained at least one of the predictable characteristics including:

1. Highly recommended HAD use: Global standard guidelines clearly and strictly recommend the use of specific drugs for the treatment of specific diseases as proven by experts and the evidence of clinical trials for risk reduction. Therefore, it makes clear a definite decision for doctors to always follow the guidelines for every patient with the same disease or condition which creates a strong association between a specific HAD and ICD10. For example, patients diagnosed with atrial fibrillation are almost always treated with aspirin to prevent life threatening complications (recommended by the 2017 ESC/EACTS Guidelines for the management of valvular heart disease^35^ and 2020 ESC Guidelines for atrial fibrillation^38^).
2. Distributive shock related condition: The medical condition leading to shock or severe tissue hypoperfusion caused by dysfunction of vascular permeability which typically requires a vasoconstrictor (Norepinephrine) to maintain blood volume and hemodynamics. Therefore, patients with shock are typically treated with vasoconstrictor agents^39^.
3. Diseases in children: Interventions in children especially infants or toddlers normally require sedative drugs (HAD) to make children cooperate^40^. Therefore, the sedative procedure means children are coincidentally related to HAD use whether or not their disease is actually related to HAD use.

According to the pattern of HAD use with standard guidelines, we explored the association between the top 5 ICD10s and HAD using specific false negative ratio (sFNR). We used sFNR to specifically measure the model prediction performance for each ICD10 (see Table 11). The lower the value of sFNR, the more the model did not miss HAD use. We manually explored 35 visits where the patient was diagnosed with a personal history of long term (current) use of anticoagulants (ICD10 with the highest HAD percent). The results showed that there were no false negative visits which led to zero sFNR. Warfarin (HAD), an anticoagulant, was commonly prescribed for some long standing cardiac diseases such as mitral stenosis, presence of prosthetic heart valve, tricuspid insufficiency, atrial fibrillation, and atrial flutter^35^ as a drug of choice. Therefore, patients with those cardiac diseases typically presented with a strong pattern of anticoagulant use for a long life span.

#### Prediction performance: IPD

The results of the IPD data at the probability cut point 0.3 showed that the model accuracy, precision, recall and F-1 score on HAD prediction were 0.69, 0.67, 1.00 and 0.80, respectively (see Fig. 6). The model evaluation showed one false negative. This false negative, comprised of 3 ICD10s (Blindness one eye, Dislocation of lens, Other senile cataract with HAD percents 11.11, 22.22, 8.51, respectively), was interpreted by HAD percent as a visit with low association of HAD use (prediction of 0.27 according to HAD percent). However, Pseudoephedrine (HAD) was prescribed in this visit. Although this visit was labeled as an actual HAD visit by definition, this visit was not a true HAD visit because the HAD prescription was unusual. Therefore, the interpretation of the inappropriateness of HAD prescription was adjusted to a true negative (aTN) as previously described in the method. Conforming with medical practice, a standard treatment of these ICD10s shows no association with Pseudoephedrine^41, 42^. We defined this event of inappropriate HAD use as a HAD–ICD10 mismatch (see the definition of HAD–ICD10 mismatch in the method section). In this case, machine learning made a correct prediction. The false prediction error occurred, due to the incidence of HAD–ICD10 mismatch. Interestingly, all of the top 5 highest HAD percentages showed zero value in specific false negative ratio which indicates the excellent performance in prediction for the high frequency HAD use diseases. Recall value also indicates the model has good ability for use as a screening tool.

#### Prediction errors

Regarding the HAD–ICD10 mismatch results in the HAD binary classification cycle, we found one true prediction error (one aFN in the 33 HAD–ICD10 mismatch as shown in Fig. 7). The cause of this prediction error was because the model was under-fitted due to too few numbers of the target ICD10s in the training and test sets. Moreover, the data set only provided drug-ICD10 association with a visit but not specifically for a single drug to ICD10. This ambiguous association created an unclear pattern that slowed the model from learning whether the HAD prescription was appropriate. Therefore, a prediction error caused by ambiguous drug-ICD10 association increased the overall error. We classified this type of error into two main characteristics: (1) Error associated complex ICD10 relationship (EACIP); (2) Error associated multi-drug use (EAMU).

#### Error associated complex ICD10 relationship (EACIP)

EACIP is a prediction error caused by insufficient machine learning processing resulting from a complicated relationship between ICD10s within the same visit and a multi-modality of management. For instance, a patient diagnosed with secondary malignant neoplasm of lung (ICD10). Diseases related to malignancy typically require cytotoxic chemotherapy (HAD) for treatment, and palliative care (ICD10), a combination of supportive treatments that usually involve following a process for the management of incurable malignancies. Drugs related to palliative care are mainly used for symptomatic treatment and less likely to relate to HAD. During the treatment process, a patient is treated with cytotoxic chemotherapy in the first visit and then later receives medication for palliative care in following visits, aiming for supportive treatment not for curing the neoplasm of lung. Therefore, the palliative care visit is classified as a non-HAD visit whereas the model predicts it as a HAD visit due to the weight prediction of cancer as a result of a false positive. This type of prediction error commonly occurs in ICD10s presenting with multi-stage treatments (such as in this case with palliative care and the treatment of malignant neoplasm of lung). This pattern of errors also generalizes to visits with other types of different disease stages such as a stage of investigating for confirming a final diagnosis, an alternative treatment for a principal disease, or checking up patient health status in a follow-up stage. This type of error occurs more often especially when there are multiple ICD10s in one visit. We analyzed this kind of ICD10 relationship by dividing ICD10s on each visit into two groups: principal ICD10 and co-morbidity. First, principal ICD10 is the primary ICD10 determined by the highest number of drugs related to the ICD10 for that visit. If a principal ICD10 is related to HAD, an average HAD percent representing the visit should be close or equal to the HAD percent of the principal ICD10. For the second group, co-morbidity is a group of other ICD10s indicated by less (co-morbidity) or no (misclassification or cured diseases) association with treatment. This relationship pattern was mainly found in multiple ICD10s visits and applicable with HAD percent to classify HAD use. However, this assumption was not always valid and caused prediction errors which we observed, with the pattern of errors presenting into two types: (1) high probability of HAD use mismatched with a non-HAD principal ICD10 (Type 1 error). This pattern occurred when high HAD percent in co-morbidity ICD10s influenced and lifted the prediction value; (2) low probability of HAD use mismatched with a HAD principal ICD10. This occurred when low HAD percent in co-morbidity ICD10s influenced and lowered the prediction value (Type 2 error). We used these two types of errors (Type 1 & 2 errors) to discuss prediction errors in false positives and false negatives in three patterns of the principal ICD10s: ICD10s related to management; ICD10s related to chief complaint; and ICD10s related to the highest number of HAD percent.

1. ICD10s related to management: An indication of HAD was varied depending on the stage of treatment. Thus, HAD percent did not always imply HAD use for the same ICD10 in every visit. The HAD use depended on disease management such as the indication of use in chemotherapy session of neoplasm, spontaneous vertex delivery, follow-up care involving removal of fracture plate and other internal fixation device, surgical operation with anastomosis, bypass or graft, or other surgical procedures.
  Example of Prediction errors in this case:
  Given the same patient in the EACIP error type section, the patient was diagnosed with secondary malignant neoplasm of lung (principal ICD10, high HAD percent) and palliative care (co-morbidity, low HAD percent). In this visit, no HAD was used because of palliative care. This case resulted in a false positive, with this scenario identified as a Type 1 error with management - ICD10 relationship. Treatment in palliative care was associated with HAD only 9% while secondary malignant neoplasm of lung was above 60%. The contradicted relationship interfered with model interpretation and caused errors in the follow-up visits when the patient received supportive care without HAD for the malignancy treatment.
2. ICD10s related to chief complaint: Generally, the principal ICD10 primarily indicated an active disease or complication while co-morbidity ICD10s indicated inactive or underlying diseases. Other patterns out of this scope might lead to a prediction error. We mostly found the errors of this type of ICD10 as a Type 1 error especially in outpatients.
  In the IPD data set, Type 1 errors were rarely found because most ICD10s in IPD related to high HAD use. However, if the principal ICD10 was non-HAD, a Type 1 error might occur. For example, a patient diagnosed with hydrocele (principal ICD10, low HAD percent), unilateral or unspecified inguinal hernia, without obstruction or gangrene (co-morbidity ICD10, high HAD percent) and hemangioma (co-morbidity ICD10, low HAD percent), were actually prescribed non-HAD (Paracetamol) because of the principal ICD10. However, the co-morbidity ICD10s had high HAD percentages, dominating the overall prediction. The model incorrectly predicted HAD use because of the influence of co-mobidity ICD10s instead of the principal ICD10.
  In contrast to the IPD results, Type 1 errors were found more often in outpatients. Type 1 errors in outpatients were typically found in visits that consisted of ICD10s related to low HAD use such as essential hypertension, type 2 diabetes mellitus without complication, and acute nasopharyngitis as principal ICD10s but the patients were also diagnosed with inactive or underlying ICD10s related to high HAD use such as atrial fibrillation, dislocation of shoulder joint which pushed up the prediction value. In some cases, we also found a Type 2 error in false negatives, such as a low prediction value for visits where the patient was diagnosed with rheumatic heart disease, unspecified (principal ICD10, high HAD percent), essential hypertension (co-morbidity ICD10, low HAD percent), disorder of lipoprotein metabolism, unspecified (co-morbidity ICD10, low HAD percent) and anemia (co-morbidity ICD10, low HAD percent) but was treated with Emthexate Tab 2.5 mg (HAD). The overall prediction of HAD use was less than usual because of the influence of the co-morbidity ICD10s as a result incorrectly predicting non-HAD instead of HAD.
3. ICD10s related to the highest HAD percent: This error occurred when there were multiple principal ICD10s within a single visit, it increased the uncertainty association of the principal ICD10s to the prediction value. Because we assumed that the indication for HAD use depended on the highest HAD percent in that visit but the ICD10 with the highest HAD percent did not always relate to the prediction of HAD use and led to prediction errors.
  Example of Prediction errors in this case:
  In the intpatient data set, we found only one such error. The model predicted high probability of HAD use in a visit where the patient was diagnosed with chronic kidney disease stage 5 (principal ICD10, high HAD percent), surgical operation with anastomosis bypass or graft (principal ICD10, low HAD percent) and other complications of cardiac and vascular prosthetic devices, implants and grafts (principal ICD10, low HAD percent). The high prediction of HAD use was influenced by the first ICD10 (Chronic kidney disease stage 5) which was logically correct. However, the patient did not receive any HADs in this visit because he was admitted for the follow-up stage of other active low HAD use principle ICD10s as described in the EACIP error type section.
  In the OPD data set, we also found a similar error pattern. The model predicted high probability of HAD use in a visit where the patient was diagnosed with chronic kidney disease stage 5 (principal ICD10, low HAD percent), chronic viral hepatitis B without delta-agent (principal ICD10, high HAD percent), essential hypertension (principal ICD10, low HAD percent), and atrial fibrillation and atrial flutter, unspecified (principal ICD10, high HAD percent) but the patient was not given any HADs. The high prediction of HAD use was influenced by the last ICD10 (atrial fibrillation and atrial flutter, unspecified) which was logically correct. Although we did not know exactly which ICD10 was associated with the prediction of HAD use in the case of multiple high HAD percent principal ICD10s, the general prediction performance had no effect because the final prediction ended up suggesting HAD use anyway.

#### Error associated multi-drug use (EAMU)

The number of drug prescriptions directly influenced HAD prediction. In general, one non-HAD prescription was logically related to low prediction of HAD use. However, when a patient was prescribed with multiple non-HADs, the non-HADs were accumulated and increased an overall prediction of HAD use. We found that visits which had non-HADs of at least 4 types significantly increased the prediction value. The accumulation of the number of non-HADs resulted in the increase of prediction value and passed the cut point leading to prediction errors. For example, a singleton born in hospital (Z380) rarely used HAD. Therefore, the model predicted low HAD use for this ICD10. However, the prediction was higher in some visits of the same ICD10 which received more than 4 types of non-HADs, and finally the accumulation effect of multiple non-HADs misled the model to misinterpret this event as a false positive.

### Cycle 2: HAD type classification cycle

After both OPD and IPD data sets with prediction cut points below 0.06 and 0.3 were excluded, the remaining data sets were passed to train and predict HAD types. The prediction results were then analyzed in the HAD type classification cycle.

#### Prediction performance

We used accuracy, precision, recall and F-1 score to measure the model performance for predicting HAD types. In practice, multiple HAD types could be used in the same visit depending on the treatment guidelines of different ICD10s. Because of this effect, we found that the average of precision was quite low in contrast with the average of recall in both data sets (see Tables 14 and 15). This characteristic of performance indicates it is good for using as a screening tool rather than as a confirmation test. However, the average of accuracy was more than 0.5 in both data sets (average accuracy in Tables 14 and 15). Furthermore, the prediction performance was better in the case of serious diseases which required precise HAD use especially in the IPD department due to being highly related to HAD use. For example, unspecified anemia unspecified thrombocytopenia, communicating hydrocephalus, tracheostomy malfunction, acute respiratory failure, systemic lupus erythematosus with involvement of organs and systems, glomerular disorders in systemic connective tissue disorders, and failed or difficult intubation were strongly associated with HAD, and found no incorrect HAD predictions. However, the overall model performance in the IPD data set was lower than the OPD data set due to the much higher proportion of non-HAD prescription data in outpatients, leading to the increased number of true negatives.

#### Prediction errors

#### Result adjustment

We also used HAD percent, a modified HAD binary classification cycle, and HAD percent indicating the frequency of HAD use for each HAD type, to readjust the misclassification results. HAD percent provides a general perspective of the amount of HAD use in the same ICD10. However, there are several limitations of this parameter as discussed earlier in the discussion in HAD binary classification cycle.

In the OPD data set, we found that BIG type showed a low proportion of adjusted false positive to false positive (3%) whereas a high proportion was found in CVS type (63% of adjusted false positive to false positive). Although ICD10s treated with BIG type were highly associated with the cardiovascular system, HAD that were classified as CVS type were rarely found in the outpatient setting. The reason behind this phenomenon was the different medications used in the different stages of diseases. Diseases prescribed with BIG type recommended treatment most likely required long term treatment while the pattern of CVS type of HAD use was sporadic. Therefore, the prediction result of BIG use was better than for CVS because of a strong and regular pattern. Some HAD use in the CVS type were used for symptomatic treatment such as using with anti-malignant drug for preventing complications from cytotoxic agents, to treat electrolyte imbalance in kidney disease such as Gitelman syndrome, where the pattern of use was irregular. Therefore, the proportion of adjusted false positive was relatively high compared with BIG type. The proportion of adjusted false positive to false positive of CNS and ANS type were 85% and 43%, respectively. These two HAD types were commonly used for symptomatic treatment which were not specific to diseases (similar to CVS). Therefore, the pattern of CNS and ANS use was irregular because the treatment could be alternatively used with other non-HADs. The proportion of Tumor type was 49%. Like BIG type, the purpose of medication use in Tumor is for a defined treatment. Immunosuppressive (anti-inflammation and microtubule inhibitor) was the most common Tumor drug type used in the outpatient setting for treating autoimmune diseases such as rheumatoid arthritis, SLE, and scleroderma. However, the proportion of Tumor was high compared with BIG even as used for defined treatment. This was because there were more alternative choices of non-HADs than diseases found in BIG type which created an irregular pattern of use and resulted in lower HAD percent and high proportion. Among all HAD types, we found only 1 adjusted false negative in Tumor type (see Fig. 13). We manually explored this false prediction to determine why the model did not predict the HAD use. The patient in this visit was diagnosed with juvenile rheumatoid arthritis (specific HAD percent was 75%). The patient was prescribed with Emthexate, an immunosuppressant, according to the standard guidelines^37^. The evidence of HAD percent and standard guidelines supported that the HAD use in this visit was appropriate. However, the probability of HAD prediction was 0.0115 (the cut point of non-HAD at P25 was 0.0117) which was interpreted as non-HAD. This prediction error was caused by the small sample size for this illness which created unreliable HAD percent, cut points, and HAD classification.

In IPD data set, Tumor type (which was the major group) presented the lowest proportion of adjusted false positives (10%) compared with the other types and much lower than the proportion found in the outpatients. Although the treatment of choice for Tumor could be different depending on the stage of tumor, patient’s condition, and guidelines, the IPD data set was mainly admitted to treat the tumor. Therefore, the drug prescription was more specific to the ICD10s than the outpatients where most visits were targeted to follow up the outcome of treatment. Because of the strong pattern between ICD10s and Tumor type for HAD, the HAD prediction was easily classified resulting in low proportion of adjusted false positive. CVS type showed a stronger disease-management pattern than the outpatients resulting in 23% of adjusted false positives. Other HAD types made up 40% to 50% of the total which were unremarkable because there were several confounding factors interfering with the HAD–ICD10 association pattern such as EACIP and EAMU.

#### Error associated with sampling process (EASP)

Apart from the errors caused by HAD–ICD10 association patterns, we also found another pattern of errors which mainly involved the sampling process. Because we treated the samples independently of each other, the selected data set of drug prescriptions might not include all prescriptions from one visit. Occasionally, the prediction errors of prescription data were identified as false positive especially when there were multiple HAD types within one visit. We later called this type of error “Error associated with sampling process” (EASP). For instance, a patient diagnosed with I340 (Mitral valve insufficiency), I350 (Aortic valve stenosis) and I48 (Atrial fibrillation and flutter) received CVS and BIG group HAD. During the sampling process, only the CVS drug prescription was selected in the testing group. The error occurred when we evaluated the BIG group for this visit. The machine learning model predicted that the patient supposed to receive BIG group because the ICD10 was highly related to the BIG group but the prediction was interpreted as a false positive because the BIG group was not selected in the test set. However, this EASP did not have much effect on the overall accuracy and model validity, and could be disregarded since we can readjust the result.

#### ICD10 percent

ICD10 percent is the percentage of visits that have a specific type of HAD prescribed out of all visits with the same specific ICD10 code. ICD10 percent shows a big picture of how the diseases are related to HAD where high ICD10 percent indicates strong association between ICD10 and HAD. The results showed that the pattern of HAD use was different between in and outpatients even for the same HAD type. The types of ICD10s found in inpatients were generally much more severe than outpatients and required specific types of HAD for treatment resulting in a high ICD10 percent (see Table 17). Conversely, diseases found in outpatients were less severe or were visits in a follow up stage which were related to supportive or symptomatic treatment which required less and irregular HAD use. Regarding BIG and Tumor types, we found those HADs were commonly prescribed in both the in and outpatients whereas other HAD types were found more commonly in inpatients. ICD10s related to BIG and Tumor types usually require long-term treatment for a life-long period which produced the similar pattern in both in and outpatients. The administration of HAD requires close monitoring and it is safer to admit a patient to be closely monitored in a hospital which leads to the strong HAD - ICD10 association only in the inpatients. In practice, we could see a lot of HAD were also prescribed in the outpatients because a hospital has limited resources and cannot accommodate all patients for an entire treatment process. Doctors then choose to discharge a stable patient to continue treatment at home and appoint the patient to follow up and review the treatment outcome at an outpatient department. This why the high ICD10 percent was also present in the outpatients.

### HAD–ICD10 mismatch

We manually explored the HAD–ICD10 mismatch cases in both validation (test sets in HAD binary classification cycle and HAD type classification cycle) and evaluation (excluded data set in HAD type classification cycle) sets and summarized three possible causes of those errors:

1. Incomplete ICD10 record: Not all proper ICD10s are entered into the system. For example, when a patient visits a hospital for a routine check-up for a chronic disease, coincidentally, they may having a common cold or feel anxiety. A doctor might prescribe Pseudoephedrine or Alprazolam for symptomatic treatment without entering the ICD10 related to those illnesses into the system.
2. Incorrect ICD10 record: An incorrect ICD10 is accidentally or intentionally entered into the system. Due to numerous ICD10s, the doctors might not be able to find a proper ICD10 which truly represents the patient’s conditions. They might choose an alternative ICD10 based on their experience, leading to an error.
3. Incorrect HAD prescription: This cause is a true HAD prescription error where a doctor accidentally or intentionally prescribes HAD which is not associated with the ICD10 which can lead to an adverse drug event.

**Figure 15:**
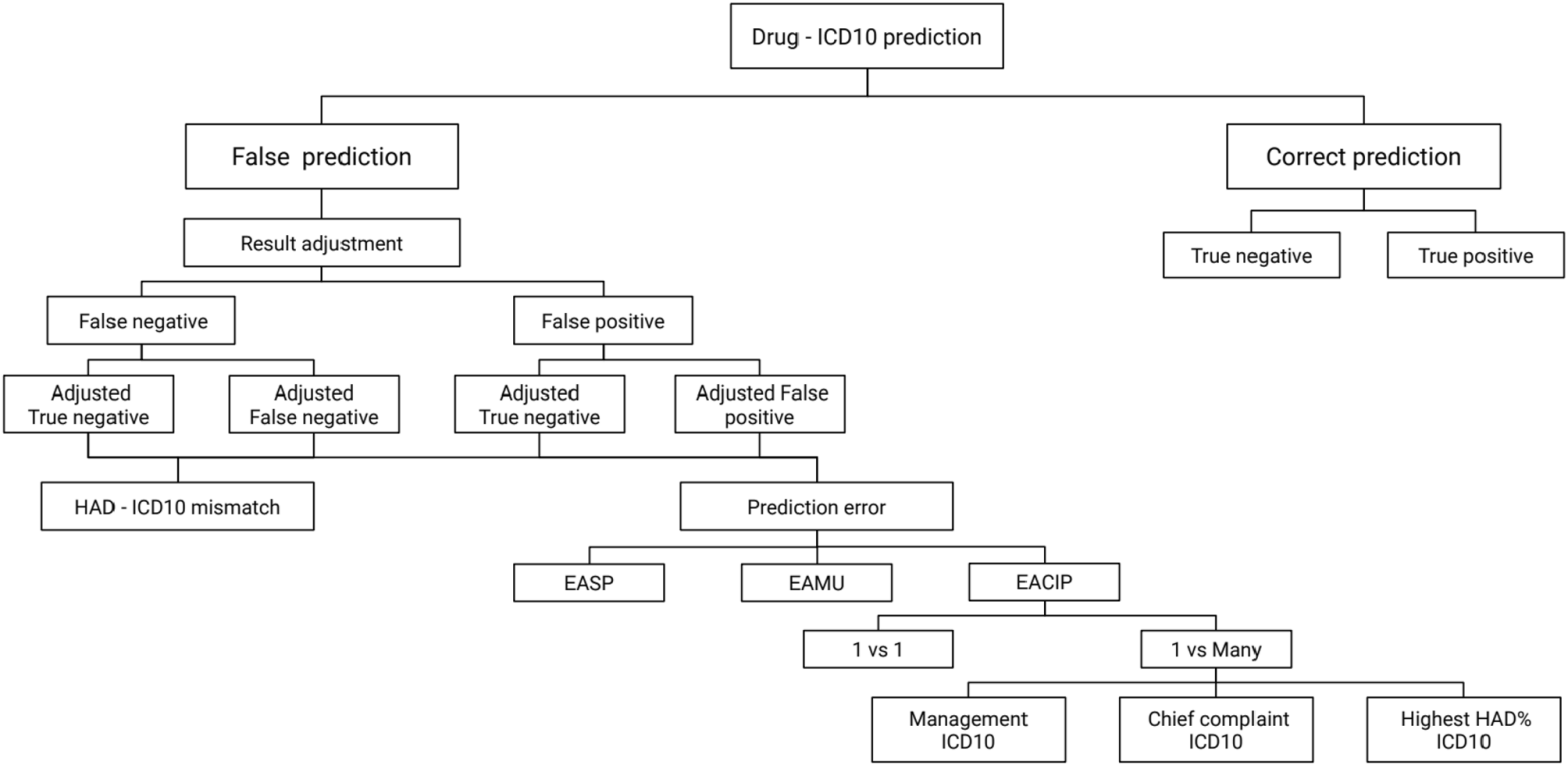
HAD prediction mind map.

Based on the input information, we were not able to distinguish the cause of errors to identify the true HAD prescription errors. In summary of the HAD screening results in inpatients, the screening protocol was able to detect 1 HAD–ICD10 mismatch in the test set and 12 HAD–ICD10 mismatches in the evaluation set of HAD binary classification cycle, no mismatches in the HAD type classification cycle, and no misclassifications across all test sets. However, our protocol in the outpatients found more inconsistencies, resulting in 32 HAD–ICD10 mismatches and 1 misclassification in the test set, 105 HAD–ICD10 mismatches and 1 misclassification in the evaluation set of the HAD binary classification cycle, and 29 HAD - ICD10 mismatches in the test set of the HAD type classification cycle without misclassification (see Table 12). As validation of the results from the outpatients, the machine learning model demonstrated a high performance by detecting 61 examples of non-associated HAD use (98%). One visit was incorrectly identified by the model because of the insufficient number of sample recruitment (discussed in detail in the limitations section). Evaluating the results from the outpatients, the machine learning model demonstrated a high performance detecting 105 cases of non-associated HAD (99%) and one HAD–ICD-10 mismatch (inappropriate use of Pseudoephedrine) which was not identified by the model. Although the screening protocol still missed some HAD - ICD10 mismatches, the process massively helped to reduce the number of cases which actually required a review of HAD use. For example in the test sets, 131 of total 38,881 cases were required for review in HAD binary classification cycle and 212 of total 47,224 cases were required for review in HAD type classification cycle. Common HAD mismatches were Pseudoephedrine, Alprazolam, Maforan, and Emthexate (see Table 12). The first two drugs were used for symptomatic treatment of nasal congestion and insomnia and usually used alongside treatment for a principal diagnosis while the other two drugs were more specifically used in a narrow scope of diagnoses. We further discussed the limitation of the study why those two HAD–ICD10 mismatch cases were not detected in the limitations section. Figure 15 summarizes the entire process of HAD screening steps. The screening process started from the machine learning model predicting HAD use. The false predictions were adjusted by HAD percent as aFP, aTP, aFN, and aTN. Part of aFN and aTN when referred to unrelated use of HAD were identified as HAD–ICD10 mismatches. All false predictions could be caused by EASP, EAMU and EACIP.

## Conclusion

Drug prescription error is an important issue in medical practice which may lead to unnecessary cost and serious complications. We developed a machine learning model, using a gradient booster classifier, and a HAD prescription screening protocol aiming to screen the appropriateness of HAD use. We divided the screening process into two cycles. In the HAD binary classification cycle, we trained the machine learning model and designed a protocol to detect visits related to HAD from non-HAD visits. We proposed a cut point selection protocol to fine-tune an appropriate threshold to gain the best screening results. In the HAD type classification cycle, we used predicted HAD visits from the HAD binary classification cycle to train new models to predict types of HAD and readjusted the results by HAD percent. Finally, machine learning was able to identify over 98% of HAD–ICD10 mismatches in the test set and 99% in the evaluation set which were actually related to prescription errors. We believe that our new approach has potential and practical use for screening HAD errors from drug prescriptions. The application has the potential benefit to reduce the manual drug verification process and precisely verify the appropriateness between high alert drug prescriptions and ICD10s.

## Limitations

There are three main limitations in our work. First, the final check of HAD–ICD10 mismatches still require a manual final check process which limits the ability to scale to a large amount of data. Because we only sampled a data set from a one year period to evaluate ADEs, this leads to the second limitation of the insufficient number of some HADs and ICD10s. With too small a number of samples, the model was insufficiently trained resulting in poor predictive performance for those ICD10s. We only used information between drugs and ICD10 to evaluate the appropriateness of HAD use. In practice, other medical information such as clinical notes, patient’s conditions, laboratory and investigation results, and radiological reports is necessary to support the decision of appropriate drug use. The integration of this additional information might help to increase the model efficacy of HAD verification. Further work is to scale this approach to a larger data set and link other medical data to improve the model efficacy. Third, the definition of HAD–ICD10 mismatch is unclear and still relies on a clinical judgment. Further study is needed to convert this judgment to a computational process to automatically detect the errors. The uncertainty zone where we excluded those prescription data that had prediction values between P25–P75 from the interpretation was not fully explored. The interpretation of HAD–ICD10 mismatches within this zone is still a challenge for future study because the pattern of HAD use is not clear. Additionally, the P25–P75 cut point selection criteria in the HAD type classification cycle was too simplified. Further research is to fine tune and develop a process that improves and personalizes prediction results for each HAD type. We hope that in the future, the improvement of screening protocols with the powerful machine learning model is applicable to implement a HAD mismatch detection tool.

## Further work

HAD screening process is a complicated task. We started with investigating the general concept of how a supervised machine learning classifier helps to screen for inappropriate use of HAD, taking the knowledge from this study to improve the screening protocol aiming at scaling, generalizing, and automating this concept to a large data set.

## Data Availability

The study protocol and source code are publicly shared upon request. Access to the data used for this study is prohibited due to patient privacy protection.

## Code availability

The source code is publicly (GNU General Public License v3.0) available at https://github.com/u4507075/icd_10 on the had branch.
